# Gga-miR-92-targeted TNFRSF1B inhibits the replication of influenza A virus by degrading TRAF3

**DOI:** 10.1128/jvi.00674-26

**Published:** 2026-06-12

**Authors:** Zhiyuan Liu, Menglu Fan, Yiran Zeng, Yiqing Zheng, Lulu Deng, Lingcai Zhao, Jun Xia, Jihui Ping

**Affiliations:** 1 MOE International Joint Collaborative Research Laboratory for Animal Health and Food Safety & Jiangsu Engineering Laboratory of Animal Immunology, College of Veterinary Medicine, Nanjing Agricultural University 70578, Nanjing, China; 2 Key Laboratory for Prevention and Control of Herbivorous Animal Diseases of the Ministry of Agriculture and Rural Affairs & Xinjiang Animal Disease Research Key Laboratory, Xinjiang Academy of Animal Sciences Institute of Veterinary Medicine, Urumchi, China; University of Freiburg, Freiburg, Germany

**Keywords:** RNA-seq, miR-92, OCT1, TNFRSF1B, TRAF3

## Abstract

Influenza viruses represent a significant threat to global public health. MicroRNAs (miRNAs), a class of small non-coding RNA molecules, play pivotal roles in regulating gene expression and have emerged as critical modulators of host-virus interactions. To investigate the functions of miRNAs during influenza virus infection, a miRNA library was constructed and sequenced using DF1 cells infected with the H9N2 influenza virus. From the differentially expressed miRNAs, we identified miR-92 as a key antiviral host factor. Mechanistically, miR-92 inhibits viral replication by targeting TNFRSF1B, which, in turn, enhances type I interferon signaling responses. Promoter region analysis demonstrated that the transcription factor OCT1 binds to the miR-92 promoter and positively regulates its transcriptional activity. Additionally, our study revealed that TNFRSF1B interacts with TNF receptor-associated factor 3 (TRAF3) and mediates TRAF3 degradation via the autophagolysosomal pathway. Specifically, TNFRSF1B facilitates the removal of K63-linked polyubiquitin chains from TRAF3. Collectively, these findings indicate that TNFRSF1B negatively regulates IFN-I responses through autophagolysosomal-mediated TRAF3 degradation, while miR-92 counteracts this inhibitory effect to exert antiviral activity. In summary, our research delineates a novel regulatory axis that modulates the interferon pathway and H9N2 influenza virus replication, providing new insights into host antiviral defense mechanisms and potential therapeutic targets.

**IMPORTANCE:**

The miR-17-92 cluster is a well-established key regulator of viral infection. However, the specific role of miR-92, an essential member of this cluster, in modulating avian influenza virus infection remains poorly defined. Here, we demonstrate that avian miR-92 exerts robust antiviral activity by directly inhibiting AIV replication. Critically, we report for the first time that the transcription factor OCT1 binds to the promoter region of miR-92 and transcriptionally regulates its expression. Mechanistically, miR-92 targets TNFRSF1B to enhance type I interferon production. Strikingly, TNFRSF1B mediates the degradation of TRAF3, thereby dampening IFN-I signaling. Together, our findings establish miR-92 as a pivotal antiviral effector during AIV infection. Beyond advancing our understanding of small RNA-mediated control of influenza virus replication, this work identifies miR-92 and its regulatory network as promising targets for the development of miRNA-based antiviral therapeutics.

## INTRODUCTION

Influenza A virus (IAV), which belongs to the Orthomyxoviridae family, is an enveloped virus characterized by a segmented negative-sense RNA genome. A key feature of IAV is its broad host tropism, enabling infection of multiple species, including humans, pigs, dogs, and various avian species (1). Seasonal influenza epidemics caused by IAV result in an estimated 290,000–650,000 deaths globally each year, representing a persistent threat to global health (2). Vaccination remains the most effective strategy for protecting susceptible populations against IAV infection. However, the virus undergoes continuous antigenic evolution through antigenic drift and antigenic shift (3), which drives the emergence of novel pandemic and epidemic strains. When vaccine strains exhibit poor matching with circulating viral variants due to rapid antigenic changes, vaccine efficacy is significantly diminished. This critical limitation underscores the urgent need for innovative IAV control approaches, such as universal vaccines targeting conserved viral epitopes or host-based antiviral strategies that target evolutionarily stable host factors.

MicroRNAs (miRNAs) are a class of evolutionarily conserved small noncoding RNAs, typically 21–23 nucleotides in length (4). Their canonical mode of action involves sequence-specific binding to the 3′ untranslated region (3′UTR) of target mRNAs, thereby mediating translational repression or mRNA degradation (5). Accumulating evidence highlights the pivotal role of miRNAs in modulating host-virus interactions. For instance, miR-451 has been shown to inhibit porcine reproductive and respiratory syndrome virus (PRRSV) replication by directly targeting the proteasome subunit PSMB8 (6). Similarly, gga-miR-30c-5p suppresses avian reovirus (ARV) replication by directly targeting ATG5, a core component of the autophagic machinery, thereby inhibiting ARV-induced autophagy (7). In the context of IAV infection, miR-200b-3p has been reported to act as a pro-viral factor by targeting TBK1 (TANK-binding kinase 1), thereby negatively regulating IRF3 and NF-κB activation (8). Gga-miR-92, a small noncoding RNA localized on chicken chromosome 1, participates in diverse biological processes by mediating the degradation of its target mRNAs (9, 10). A recent study further demonstrated that miR-92a antagonizes mouse mammary tumor virus (MMTV) replication by directly targeting viral genomic RNA (11). Despite these insights, the specific roles of miR-92 in IAV infection, particularly its regulatory effects on viral replication, host antiviral signaling, and the underlying molecular mechanisms, remain largely unknown.

Tumor necrosis factor receptor superfamily member 1B (TNFRSF1B), also commonly referred to as TNFR2, is a type I transmembrane receptor that exhibits a high affinity for tumor necrosis factor alpha (12). Upon binding to TNF-α, TNFR2 undergoes conformational changes to activate downstream signaling cascades, including the NF-κB and MAPK pathways (13). This molecular interaction is also involved in various physiological and pathological processes, including inflammatory responses and immune regulation (14). Previous studies have demonstrated that in specific cellular models, TNFRSF1B can transduce apoptotic signals and potentiate TNFR1-induced cell death, highlighting its dualistic role in cell fate regulation(15). TNFRSF1B has been shown to recruit the adaptor protein TNF receptor-associated factor 2 (TRAF2) and induce its K48-linked ubiquitination, leading to proteasomal degradation of TRAF2 (16). Emerging evidence links TNF superfamily signaling to influenza virus pathogenesis. For example, mice deficient in both TNF and IL-1 receptors exhibit significantly reduced morbidity and mortality following infection with the highly pathogenic H5N1 influenza virus, suggesting that TNF-mediated signaling pathways contribute to viral pathogenesis (17). These findings imply that TNFRSF1B may play a critical regulatory role in influenza virus infection, potentially through modulating host immune responses or viral replication.

H9N2 avian influenza viruses are widely prevalent in poultry and pose considerable public health risks owing to their high zoonotic potential and frequent genetic reassortment with other influenza viruses (18). Although several studies have investigated the mechanisms by which human miRNAs regulate influenza virus replication, research focusing on how avian miRNAs control avian influenza virus replication remains relatively limited. DF-1 cells represent a well-characterized avian fibroblast cell line that is widely used to study avian influenza virus replication and host innate immune responses (19, 20). Accordingly, we used DF-1 cells and H9N2 virus as the principal *in vitro* model to uncover the miRNA-mediated regulatory mechanism underlying avian influenza virus infection. In the present study, we first characterized miRNA expression profiles in DF-1 cells following infection with H9N2 avian influenza virus. Among the differentially expressed miRNAs, gga-miR-92 was found to be significantly upregulated compared to mock-infected cells. Functional assays revealed that gga-miR-92 exerts an antiviral effect by directly targeting TNFRSF1B, thereby suppressing influenza virus replication. Notably, mechanistic investigations demonstrated that miR-92 expression is regulated by the OCT1 transcription factor. Furthermore, co-immunoprecipitation (Co-IP) experiments demonstrated an interaction between TNFRSF1B and TRAF3. Subsequent western blot analyses and autophagy inhibitor treatment experiments revealed that TNFRSF1B mediates TRAF3 degradation via an autophagy-dependent pathway. In summary, these findings establish that gga-miR-92 plays a pivotal role in the host antiviral response against influenza virus infection.

## RESULTS

### Transcriptome sequencing identified host miRNAs involved in H9N2 influenza virus replication

**Fig 1:**
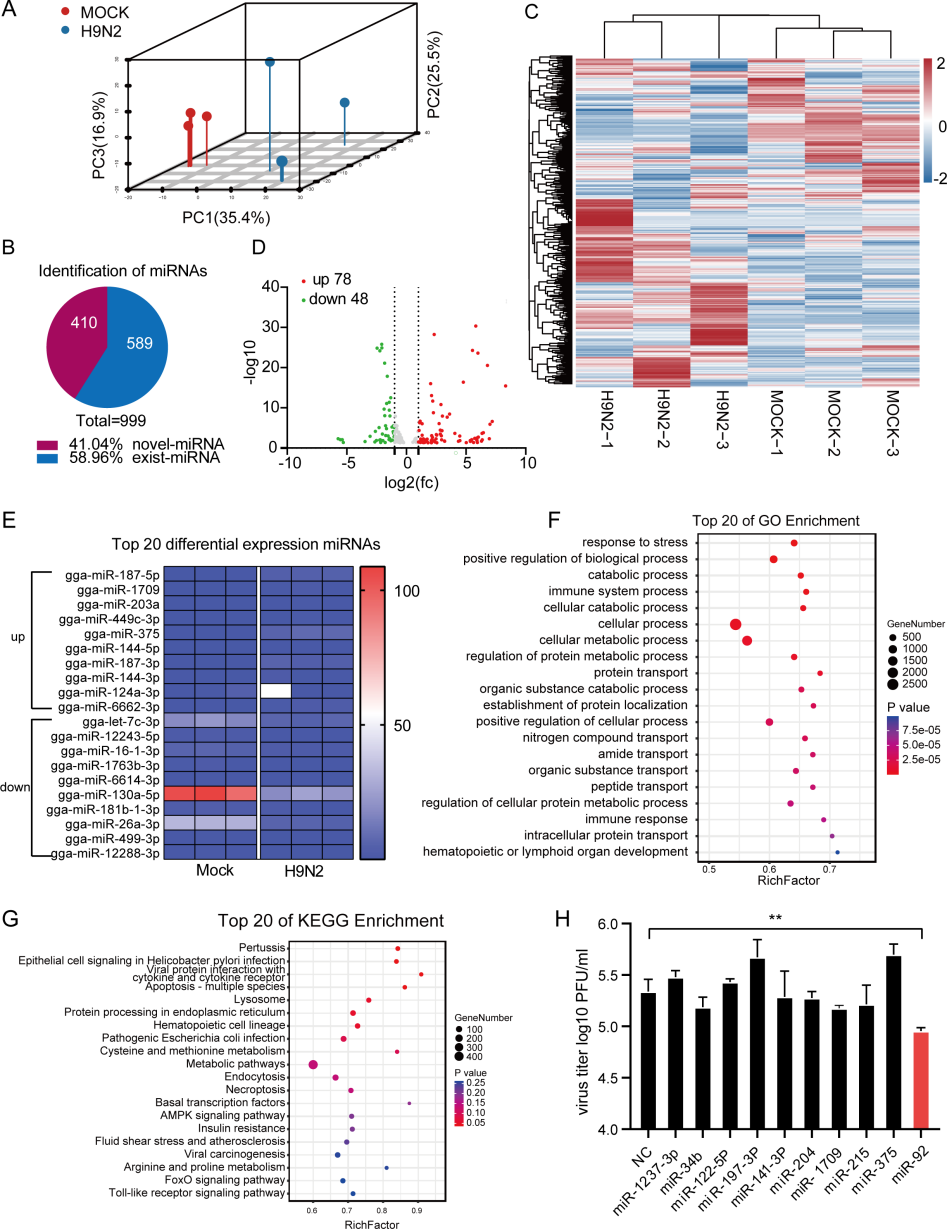
Analysis of miRNA expression profiles in DF1 cells infected with H9N2 subtype influenza virus. (**A**) Principal component analysis (PCA) of samples from H9N2-infected and uninfected groups. (**B**) Pie chart illustrating the number of identified miRNAs in H9N2 virus-infected DF1 cells. (**C**) miRNA clustering analysis of individual samples. (**D**) Volcano plot analysis of differentially expressed miRNAs. (**E**) Heatmap analysis of the top 20 DE miRNAs. (**F**) Gene Ontology (GO) enrichment analysis of the DE miRNAs. The x-axis represents the rich factor (ratio of differentially expressed genes to all annotated genes in each GO term). Bubble size corresponds to the number of enriched genes in each term. Bubble color indicates the statistical significance of enrichment (−log10(*P*-value)), with *P* < 0.05 as the threshold for significant enrichment. (**G**) Kyoto Encyclopedia of Genes and Genomes (KEGG) pathway analysis of the top miRNAs. The x-axis represents the rich factor (ratio of differentially expressed genes to total annotated genes in each pathway). Bubble size corresponds to the number of enriched genes in each pathway. Bubble color indicates the statistical significance of enrichment (−log10(*P*-value)), with *P* ≤ 0.05 set as the threshold for significance. (**H**) DF1 cells were separately transfected with 10 distinct miRNA mimics. At 24 h post-transfection (hpt), the cells were infected with H9N2 influenza virus at a multiplicity of infection (MOI) of 1. At 24 h post-infection (hpi), the cell supernatants were harvested, and virus titers were quantified via plaque assay. Data shown are the mean ± SD from three independent experiments. All statistical analyses were performed using unpaired two-tailed *t*-tests. **, *P* < 0.01.

To comprehensively explore whether influenza virus infection elicits alterations in the host miRNA expression profiles, a high-throughput miRNA sequencing analysis was performed on DF1 cells infected with the H9N2 influenza virus, with uninfected DF1 cells included as a parallel control group. To ensure the reliability of downstream analysis, principal component analysis (PCA) was first conducted to assess inter-sample reproducibility. As vividly depicted in Fig. 1A, biological replicates of each group clustered closely together, demonstrating excellent within-group reproducibility. Subsequently, a total of 999 distinct miRNAs were precisely identified through RNA sequencing technology. Among them, 589 were annotated as known miRNAs, and 410 were identified as novel miRNAs (Fig. 1B). To further visualize global differences in miRNA expression patterns between groups, hierarchical clustering analysis was performed. The heatmap clearly delineated two major clusters corresponding to the H9N2-infected and uninfected groups, with distinct miRNA expression signatures between the two cohorts (Fig. 1C).

To further identify differentially expressed miRNAs, the edgeR software was utilized. miRNAs with a fold change greater than two and an adjusted *P*-value less than 0.05 were strictly defined as differentially expressed miRNAs (DE miRNAs). As presented in Fig. 1D, comparative analysis revealed that 126 DE miRNAs were identified in H9N2-infected cells relative to uninfected controls, 78 miRNAs were significantly upregulated, while 48 were downregulated. To highlight the most significantly altered miRNAs, Fig. 1E specifically showed 10 miRNAs with the most pronounced upregulation and 10 with the most significant downregulation, which are likely to play crucial roles in the cellular response to H9N2 infection.

To elucidate the biological relevance of the identified DE miRNAs, GO enrichment analysis and the Kyoto Encyclopedia of Genes and Genomes (KEGG) pathway enrichment analysis were performed. The enrichment results, as shown in Fig. 1F and G, strikingly demonstrated that the DE miRNAs were associated with vital signaling pathways, such as the immune system process, necroptosis, and Toll-like receptor signaling pathway. These findings strongly suggest that the differentially expressed miRNAs may be deeply involved in immune regulation during the viral infection process.

Given the enrichment of DE miRNAs in immune and antiviral pathways, we hypothesized that specific DE miRNAs might directly regulate influenza virus replication. To further screen and narrow down candidate miRNAs, we collected publicly available small RNA sequencing data of avian cells infected with two additional H9N2 strains: A/environment/1/18/2007 (H9N2) and A/duck/Nanjing/06/2003 (H9N2). Specifically, the sequencing data set for A/duck/Nanjing/06/2003 (H9N2) was obtained from the NCBI GEO database under the accession number GSE147658, and the raw data for A/environment/1/18/2007 (H9N2) were retrieved from a previous study (21). Differential expression analysis was conducted for these two datasets, with the results presented in Fig. S1A and B. We further integrated the differentially expressed miRNAs induced by these three independent H9N2 strains via intersection analysis (Fig. S1C). After excluding miRNAs with well-characterized roles in viral infection, we finally selected 10 candidate miRNAs for preliminary experimental validation. Each miRNA mimic was separately transfected into DF1 cells. Subsequently, the transfected DF1 cells were infected with the H9N2 avian influenza virus. At 24 h post-infection (hpi), cell supernatants were collected, and viral titers were quantified using the plaque assay. Notably, as clearly shown in Fig. 1H, among these miRNAs, miR-92 was found to have a significant inhibitory effect on H9N2 virus replication compared to the negative control group, suggesting its potential as a key regulator in the antiviral defense mechanism against H9N2 influenza virus.

### MIR-92 inhibits H9N2 influenza virus replication

**Fig 2:**
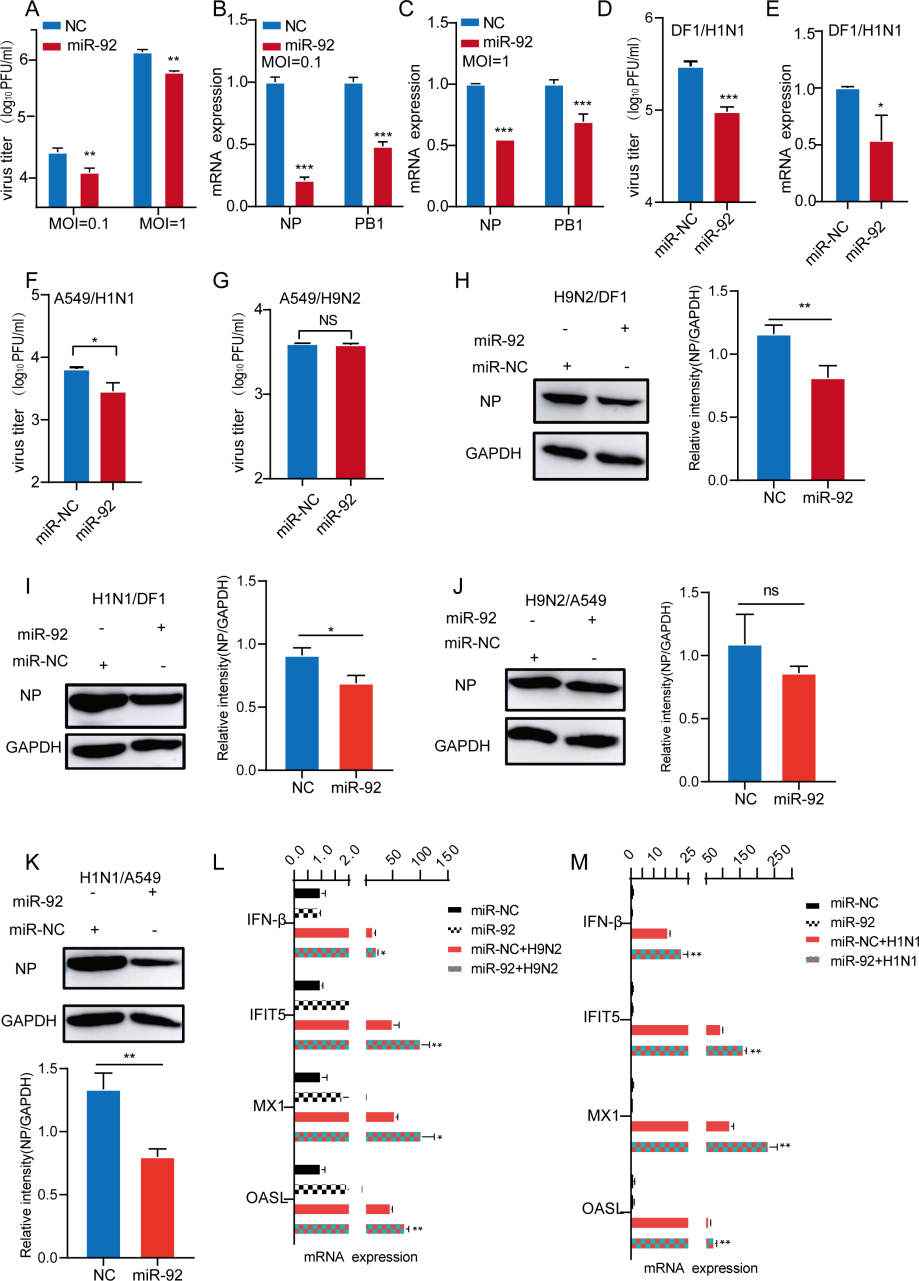
MiR-92 inhibits influenza virus replication. (**A**) DF1 cells were transfected with miR-92 mimic, with a negative control (NC) mimic as the control. At 24 h post-transfection (hpt), cells were infected with the H9N2 subtype influenza virus at an MOI of 0.1 or 1. At 24 hpi, cell supernatants were harvested, and virus titers were quantified via plaque assay. (**B and C**) The mRNA expression levels of the viral NP and PB1 genes were analyzed in miR-92-overexpressing cells. (**D and E**) DF1 cells were transfected with miR-92 mimic, with NC mimic as the reference. At 24 hpt, the cells were infected with the H1N1 subtype influenza virus. At 24 hpi, the cell supernatants and cellular samples were collected. Virus titers and viral RNA expression were determined by plaque assay and q-PCR, respectively. (**F and G**) A549 cells were transfected with miR-92 mimic, with NC mimic as the reference. At 24 hpt, the cells were infected with the H1N1 subtype or the H9N2 subtype influenza virus at an MOI of 1, and virus titers were quantified via plaque assay. (**H–K**) A549 or DF1 cells were transfected with miR-92 mimic, with NC mimic as the reference. At 24 hpt, cells were infected with the H1N1 subtype or the H9N2 subtype influenza virus, respectively. Viral NP protein was detected by western blotting. Quantification of three independent Western blot replicates is presented in Fig. S3A through S3D. (**L and M**) DF1 cells were transfected with miR-92 mimic, with NC mimic as the reference. At 24 hpt, the cells were infected with H1N1 subtype or H9N2 subtype influenza virus. At 24 hpi, cellular samples were collected, and the expression of type I interferons and interferon-stimulated genes (ISGs) was analyzed by qPCR. Data shown are the mean ± SD from three independent experiments. Western blot results are representative of three independent experiments. All statistical analyses were performed using unpaired two-tailed *t*-test or one-way ANOVA. *, *P* < 0.05, **, *P* < 0.01, ***, *P* < 0.001.

To further validate the role of miR-92 in influenza virus infection, DF1 cells were transfected with miR-92 mimics. At 24 h post-transfection (hpt), the cells were infected with H9N2 subtype IAV at a multiplicity of infection (MOI) of 1 or 0.1. Viral titers in the culture supernatants were quantified via plaque assay. The results demonstrated that miR-92 could significantly inhibit the infection of H9N2 influenza virus at both MOIs (0.1 and 1) (Fig. 2A). To verify this antiviral activity, qPCR was performed to assess the mRNA levels of viral nucleoprotein (NP) and polymerase basic 1 (PB1). At 24 h post-infection (hpi), the mRNA levels of NP and PB1 were significantly downregulated in miR-92-transfected DF1 cells compared to those in cells transfected with the negative control (NC) mimic (Fig. 2B and C). To determine whether the antiviral effect of miR-92 is subtype-specific, DF1 cells transfected with miR-92 mimics were infected with H1N1 subtype IAV at an MOI of 1. The plaque assay indicated that miR-92 could significantly inhibit H1N1 virus infection (Fig. 2D). Q-PCR experiments revealed that miR-92 could effectively inhibit the RNA synthesis of the H1N1 subtype influenza virus (Fig. 2E). Furthermore, we investigated the antiviral effect of miR-92 in human A549 cells. Interestingly, miR-92 effectively inhibited the replication of influenza A virus (IAV) H1N1 subtype, yet failed to suppress the replication of the IAV H9N2 subtype in A549 cells (Fig. 2F and G). This observation may reflect inherent subtype-specific characteristics of influenza viruses. In line with the above viral titer data, western blot analysis demonstrated that miR-92 repressed the nucleoprotein expression of both H9N2 and H1N1 subtypes in DF1 cells, but exerted no inhibitory effect on H9N2 nucleoprotein expression in A549 cells (Fig. 2H through K). Collectively, these data demonstrate that miR-92 exerts an inhibitory effect on the replication of influenza A viruses, including H9N2 and H1N1 subtypes.

### MIR-92 promotes IFN and ISG production to restrict influenza virus infection

Our previous findings established that miR-92 exerts an inhibitory effect on the replication of influenza A viruses in DF1 cells. To delineate the underlying molecular mechanism, we focused on the type I interferon pathway. Prior research has reported that miR-92 can suppress the replication of feline panleukopenia virus by potentiating the expression of type I interferon (22). Inspired by this finding, we postulated whether miR-92 could modulate the type I interferon response during influenza virus infection. To experimentally test this hypothesis, we investigated the impact of miR-92 on the transcriptional regulation of type I interferon and interferon-stimulated genes (ISGs). DF1 cells were transfected with miR-92 mimics. Subsequently, these transfected cells were challenged with H9N2 or H1N1 IAV at an MOI of 1. The transcriptional levels of IFN-β and three functionally critical ISGs (IFIT5, OASL, and Mx1) were quantified via q-PCR. As vividly depicted in Fig. 2L and M, upon exposure to either H9N2 or H1N1 influenza virus, the overexpression of miR-92 led to a significant upregulation of IFN-β and its downstream interferon-stimulated genes, such as IFIT5, OASL, and Mx1. Collectively, these data strongly support the conclusion that miR-92 plays an integral role in regulating the type I interferon response in DF1 cells during IAV infection.

### H9N2 influenza virus infection upregulates both the promoter activity and expression of MIR-92

**Fig 3:**
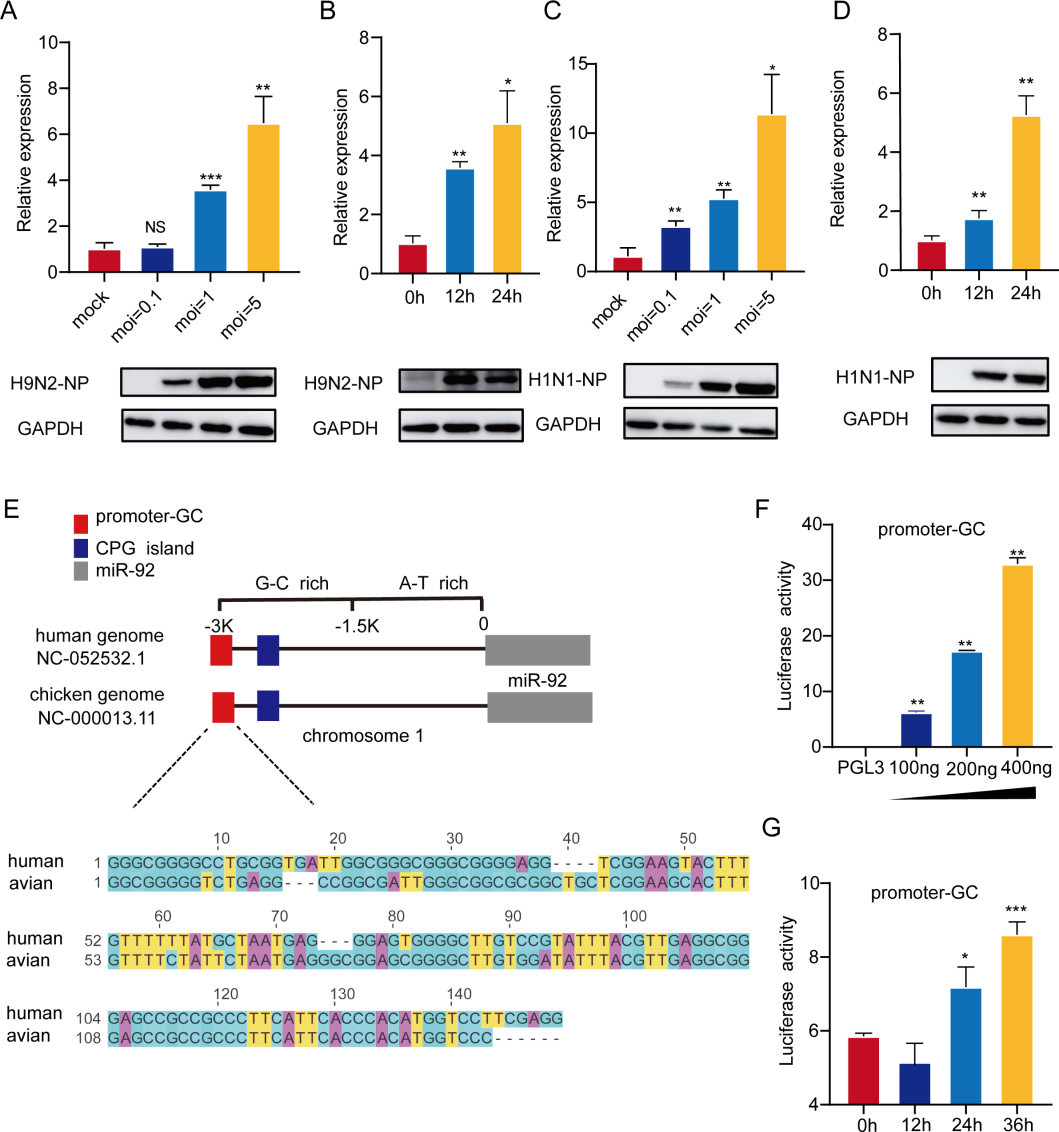
Influenza virus infection regulates both the promoter activity and expression of miR-92. (**A**) DF1 cells were infected with H9N2 avian influenza virus at different MOIs. At 24 hpi, the cells were collected, and the expression level of miR-92 was analyzed by q-PCR. (**B**）DF1 cells were infected with H9N2 avian influenza virus at an MOI of 1. At 0, 12, and 24 h post-infection, cells were harvested, and miR-92 expression levels were determined by qPCR. (**C**) DF1 cells were infected with the H1N1 subtype influenza virus at different MOIs. At 24 hpi, cells were collected, and the expression level of miR-92 was analyzed by q-PCR. (**D**) DF1 cells were infected with H1N1 avian influenza virus at an MOI of 1. At 0, 12, and 24 h post-infection, cells were harvested, and miR-92 expression levels were determined by qPCR. (**E**) Sequence analysis of the miR-92 promoter. Red represents promoter sequences in GC-rich regions, blue represents CpG islands, and gray represents miR-92. (**F**) DF1 cells were transfected with the miR-92 promoter reporter plasmid in a dose-dependent manner. The promoter activity was analyzed using the dual-luciferase reporter system. (**G**) DF1 cells were transfected with the miR-92 promoter reporter plasmid. At 24 hpt, cells were infected with H9N2 avian influenza virus. At 12, 24, and 36 hpi, cells were collected, and miR-92 promoter activity was analyzed using the dual-luciferase reporter assay. Data shown are the mean ± SD from three independent experiments. All statistical analyses were performed using an unpaired two-tailed *t*-test.*, *P* < 0.05, **, *P* < 0.01, ***, *P* < 0.001.

To comprehensively explore the regulatory effect of the influenza virus on miR-92, we first evaluated the dynamic changes in miR-92 levels using qPCR. The results revealed that following infection with H9N2 or H1N1 subtype influenza virus, the expression level of miR-92 in DF1 cells was markedly upregulated. To confirm this regulatory effect, DF1 cells were infected with H9N2 or H1N1 subtype influenza virus at escalating MOIs (0.1, 1, and 5). Consistent with the initial observation, miR-92 expression increased in a dose-dependent manner and in a time-dependent manner (Fig. 3A through D). The western blot analysis of viral nucleoprotein (NP) expression (shown beneath Fig. 3A through D) further confirmed that influenza virus replicates efficiently in a time- and dose-dependent manner. Collectively, these data establish that infection with H9N2 or H1N1 subtype IAV significantly enhances the expression level of mature miR-92 in DF1 cells. Subsequently, to elucidate the underlying molecular mechanism by which influenza virus upregulates the expression level of miR-92, we performed an *in silico* analysis of the miR-92 genomic locus. As illustrated in Fig. 3E, miR-92 is located on chromosome 1, and there are two crucial regions upstream of gga-miR-92. Notably, one of these regions is a conserved sequence within a GC-rich domain, which bears a high resemblance to the core promoter region of the human miR-17-92 gene cluster. Existing studies have reported that multiple transcription factors can delicately regulate the expression of the miR-17-92 cluster by specifically binding to its promoter region. Sequence analysis indicated that the GC-rich domain sequence is highly conserved between humans and avians. To experimentally validate the promoter activity of miR-92, we amplified this (−3,000 to −2,500 bp) GC-rich fragment from DF1 cell genomic DNA and cloned it into the pGL3-basic vector. The plasmid pGL3-miR-92 or empty pGL3-basic vector (negative control) was co-transfected with a Renilla luciferase plasmid into DF1 cells. At 36 hpt, luciferase activity was measured using a dual-luciferase reporter assay system. The results showed that the relative luciferase activity of pGL3-miR-92 was ~30-fold higher than that of the empty vector, confirming that the GC-rich region possesses strong promoter activity (Fig. 3F). Further experiments demonstrated that compared with the mock-infected control group, the luciferase activity of pGL3-miR-92 was significantly elevated in H9N2 AIV-infected cells (Fig. 3G). This result further confirmed that the miR-92 promoter is located in the GC-rich region upstream of miR-92, and infection with influenza virus can effectively enhance the activity of the miR-92 promoter. This finding is highly consistent with our previous conclusion that the expression of miR-92 is upregulated in DF1 cells after influenza virus infection. In summary, we speculate that the influenza virus may promote the activity of the miR-92 promoter, driving the transcriptional upregulation of miR-92.

### The transcription factor OCT1 regulates both the promoter activity and expression of MIR-92

**Fig 4:**
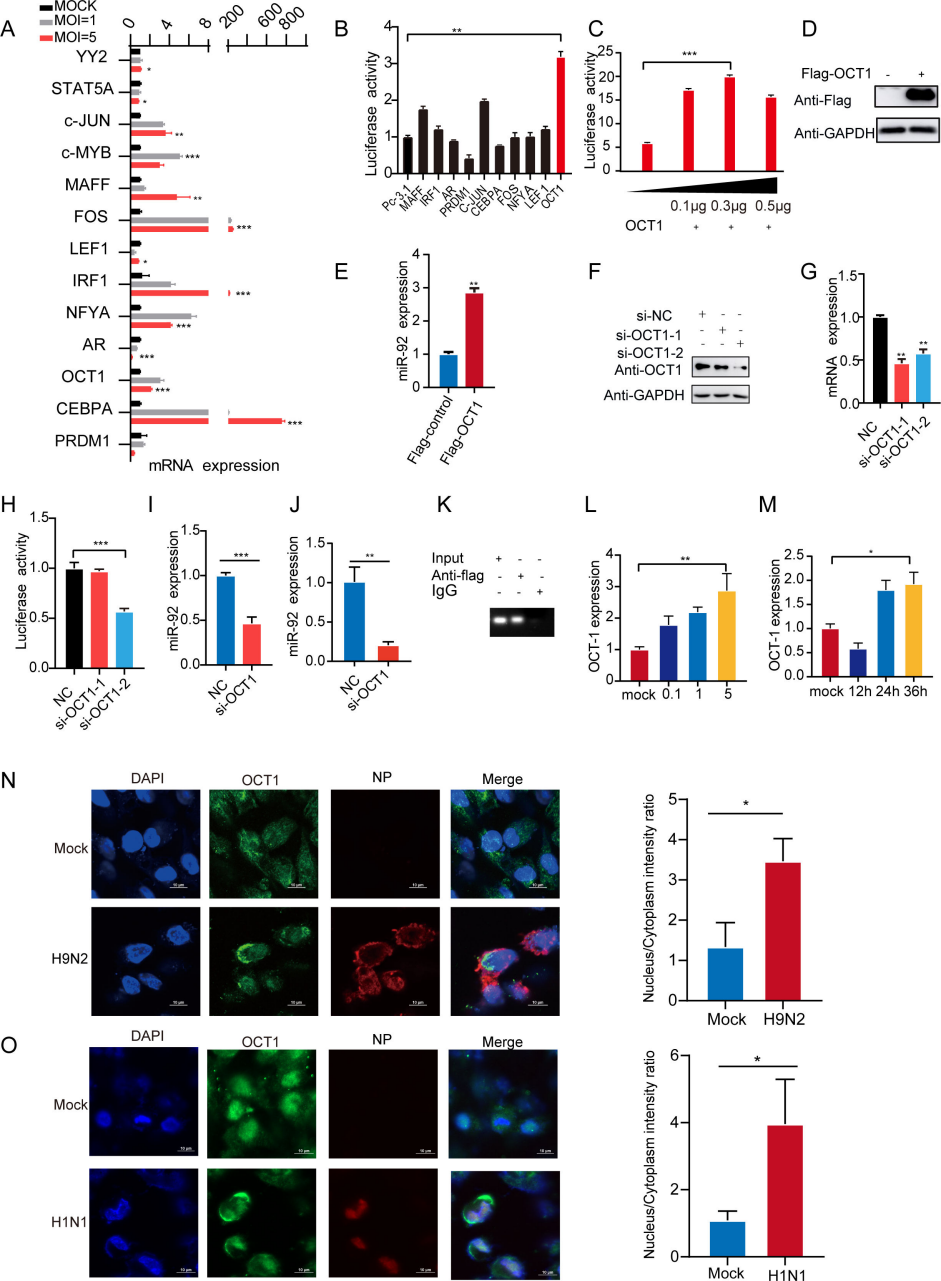
Transcription factor OCT1 regulates both the promoter activity and expression of miR-92. (**A**) DF1 cells were infected with H9N2 subtype influenza virus at MOIs of 1 or 5. At 24 hpi, cellular samples were harvested, and the mRNA expression levels of candidate transcription factors were quantified via q-PCR. (**B**) DF1 cells were co-transfected with the miR-92 promoter reporter plasmid and eukaryotic expression plasmids encoding candidate transcription factors. At 24 h post-transfection, cells were lysed, and miR-92 promoter activity was assessed using the dual-luciferase assay. (**C**) DF1 cells were co-transfected with the miR-92 promoter reporter plasmid and OCT1 expression plasmid in a dose-dependent manner. Following transfection, cells were infected with the H9N2 virus. MiR-92 promoter activity was assessed via the dual-luciferase assay. (**D and E**) DF1 cells were transfected with the FLAG-tagged OCT1 plasmid, and OCT1 protein expression was detected by western blotting. The expression level of miR-92 was quantified by q-PCR. (**F and G**) DF1 cells were transfected with OCT1 small interfering RNA (siRNA). At 24 hpt, cellular samples were harvested, and OCT1 mRNA and protein levels were analyzed by q-PCR and WB, respectively. (**H**) DF1 cells were co-transfected with the miR-92 promoter reporter plasmid and OCT1 siRNA. At 24 hpt, luciferase activity was assessed using the dual-luciferase assay. (**I**) DF1 cells were transfected with OCT1 siRNA. At 24 hpt, cellular samples were harvested, and the expression level of miR-92 was quantified by q-PCR. (**J**) DF1 cells were transfected with OCT1 siRNA. At 24 h post-transfection, cells were infected with H9N2 avian influenza virus at an MOI of 1. Cellular samples were then collected, and miR-92 expression levels were quantified by qPCR. (**K**) DF1 cells were transfected with the FLAG-OCT1 plasmid and cultured for 36 h prior to chromatin immunoprecipitation (ChIP) assays. Input DNA was used as a positive control to indicate chromatin integrity, and normal IgG was included as a negative control for non-specific immunoprecipitation. Subsequent ChIP-PCR analyses were performed to amplify the region in the miR-92 promoter that contained OCT1 binding sites. (**L and M**) OCT1 mRNA expression in DF1 cells was analyzed following H9N2 virus infection at different doses or at various time points post-infection. (**N and O**) DF1 cells were infected with H9N2 or H1N1 subtype influenza virus. At 24 hpi, OCT1 protein localization was detected by immunofluorescence assay. The nuclear/cytoplasmic (N/C) ratio of OCT1 protein was quantified with ImageJ. Additional representative immunofluorescence images are shown in Fig. S3E. Data shown are the mean ± SD from three independent experiments. All statistical analyses were performed using an unpaired two-tailed *t*-test or one-way ANOVA. *, *P* < 0.05, **, *P* < 0.01, ***, *P* < 0.001.

To further investigate the critical transcription factors involved in the transcriptional regulation of miR-92, potential transcription factors that could bind to the miR-92 promoter region were predicted using the Animal Transcription Factors Database (23). As illustrated in Fig. 4, 13 candidate transcription factors were selected, and their expression levels in H9N2-infected DF1 cells were detected via qPCR. Genes with significantly altered expression were identified as candidate transcription factors governing miR-92 expression. To determine which candidate genes directly regulate miR-92 promoter activity, eukaryotic expression plasmids for these candidate transcription factors were constructed and co-transfected with the miR-92 promoter reporter plasmid into DF1 cells. Dual-luciferase reporter assays revealed that overexpression of OCT1 led to a significant upregulation of miR-92 promoter activity (Fig. 4B). As shown in Fig. 4C, compared with the empty vector control group, overexpression of OCT1 in DF1 cells significantly enhanced miR-92 promoter activity in a dose-dependent manner. To further validate the regulatory role of OCT1 in miR-92 expression, the overexpression efficiency of OCT1 was verified (Fig. 4D). Q-PCR analysis further showed that OCT1 overexpression significantly elevated mature miR-92 levels (Fig. 4E). Subsequently, the knockdown efficiency of OCT1 protein was confirmed by qPCR and western blot analysis (Fig. 4F and G). We then co-transfected OCT1 siRNA or si-NC with pGL3-miR-92 into DF1 cells. As shown in Fig. 4H and I, OCT1 knockdown significantly decreased both miR-92 promoter activity and miR-92 expression. Moreover, OCT1 knockdown also inhibited miR-92 expression in the context of influenza A virus infection (Fig. 4J). In parallel, we constructed a miR-92 promoter mutant plasmid carrying a mutated OCT1 binding site. DF1 cells were transfected with the PGL3-GC-promoter-MUT plasmid and then infected with H9N2 influenza A virus. The promoter activity of the mutant construct was measured at multiple time points post-infection. As shown in Fig. S1D, mutation of the OCT1 binding site completely abrogated the H9N2 infection-induced upregulation of miR-92 promoter activity. These data confirm that OCT1 is required for maintaining miR-92 transcription. To directly demonstrate that OCT1 binds to the miR-92 promoter, we performed a chromatin immunoprecipitation (ChIP) assay. ChIP-PCR confirmed that OCT1 can bind to the miR-92 promoter (Fig. 4K). Collectively, these data establish that OCT1 functions as a critical transcription factor regulating miR-92 expression in DF1 cells. Given our observations that H9N2 influenza A virus infection induces miR-92 expression and that OCT1 is a regulator of miR-92, we investigated the impact of influenza infection on OCT1 expression in DF1 cells. Our findings confirmed that during avian influenza virus infection, the transcriptional level of OCT1 was significantly upregulated in a time-dependent and dose-dependent manner (Fig. 4L and M). Confocal microscopy experiments demonstrated that viral infection promoted the nuclear translocation of OCT1 protein (Fig. 4N and O). These results indicate that upon DF1 cells, influenza virus drives the transcription of OCT1, thereby facilitating the expression of miR-92.

### MIR-92 directly targets 3′ UTR of TNFRSF1B mRNA

**Fig 5:**
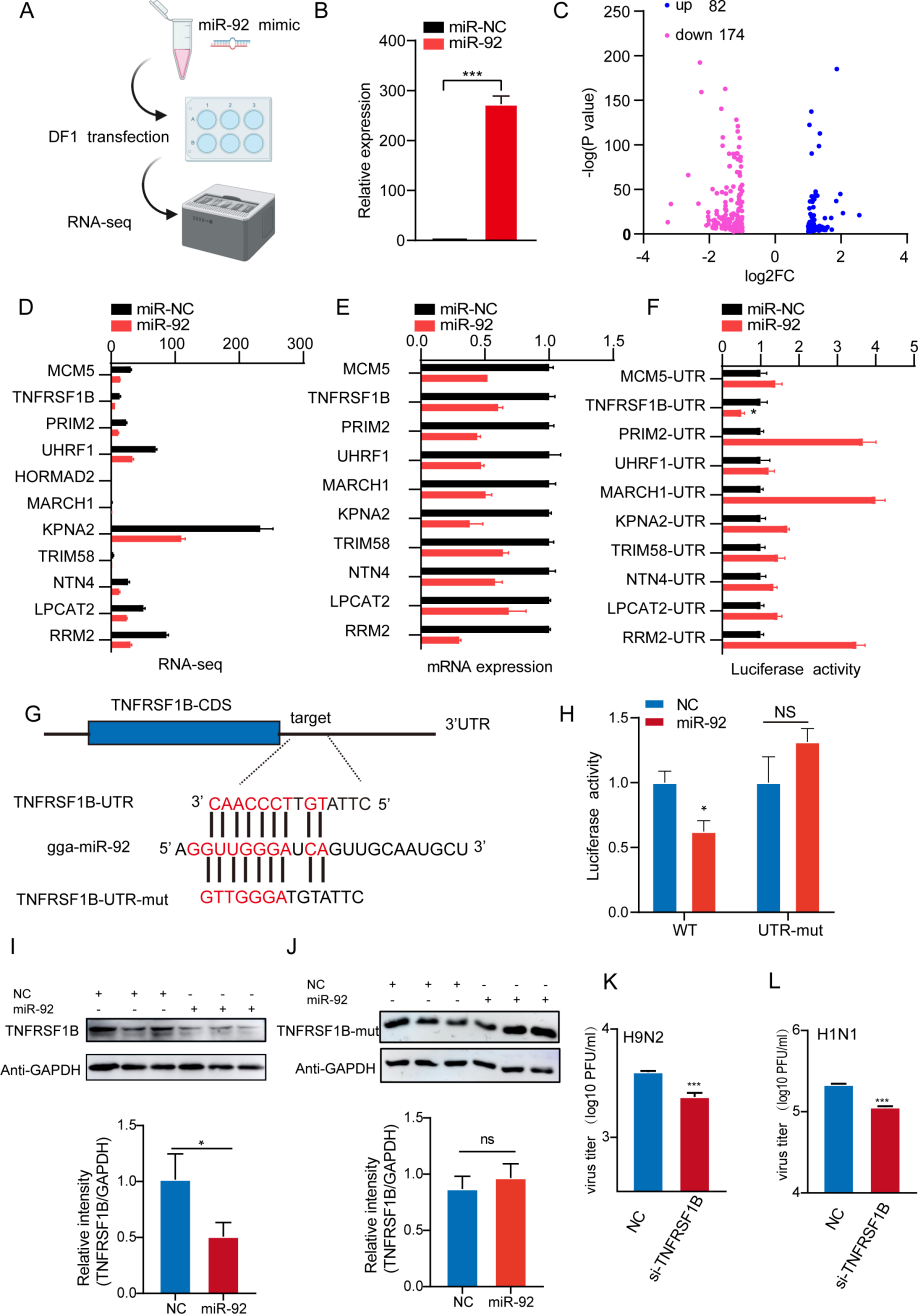
MiR-92 directly targets 3′ UTR of TNFRSF1B mRNA. (**A**) DF1 cells were transfected with miR-92 mimic. At 36 hpt, cells were harvested for RNA sequencing. (**B**) The overexpression efficiency of miR-92 was quantified by q-PCR. (**C**) Volcano plot illustrating differentially expressed genes in miR-92-overexpressing cells. Among them, 82 upregulated genes and 174 downregulated genes were significantly differentially expressed. (**D**) mRNA expression levels of candidate target genes were determined via RNA sequencing in DF1 cells overexpressing miR-92. (**E**) mRNA expression levels of candidate target genes were quantified by q-PCR in DF1 cells overexpressing miR-92. (**F**) 293T cells were co-transfected with miR-92 mimic and luciferase reporter plasmids harboring the 3' untranslated region (UTR) of candidate target genes. Luciferase activity was assessed using the dual-luciferase assay. (**G**) Sequence analysis of the binding region between miR-92 and the 3′ UTR of TNFRSF1B. (**H**) 293T cells were co-transfected with miR-92 mimic together with either the wild-type (WT) or mutant (MUT) TNFRSF1B-3′ UTR luciferase reporter plasmid. Luciferase activity was then measured using a dual-luciferase reporter assay system. (**I**) DF1 cells were co-transfected with miR-92 mimic and the FLAG-tagged TNFRSF1B-3′ UTR plasmid. TNFRSF1B protein expression was detected by western blotting. (**J**) DF1 cells were co-transfected with miR-92 mimic and the FLAG-tagged TNFRSF1B-3′ UTR mutant plasmid. TNFRSF1B protein expression was detected by Western blotting. (**K and L**) DF1 cells were transfected with TNFRSF1B-specific small interfering RNA (siRNA); at 24 h post-transfection, the cells were infected with H9N2 or H1N1 influenza virus. Viral titer was determined via plaque assay. Data shown are the mean ± SD from three independent experiments. Western blot results are representative of three independent experiments. All statistical analyses were performed using an unpaired two-tailed *t*-test or one-way ANOVA. *, *P* < 0.05, ***, *P* < 0.001.

**Fig 6:**
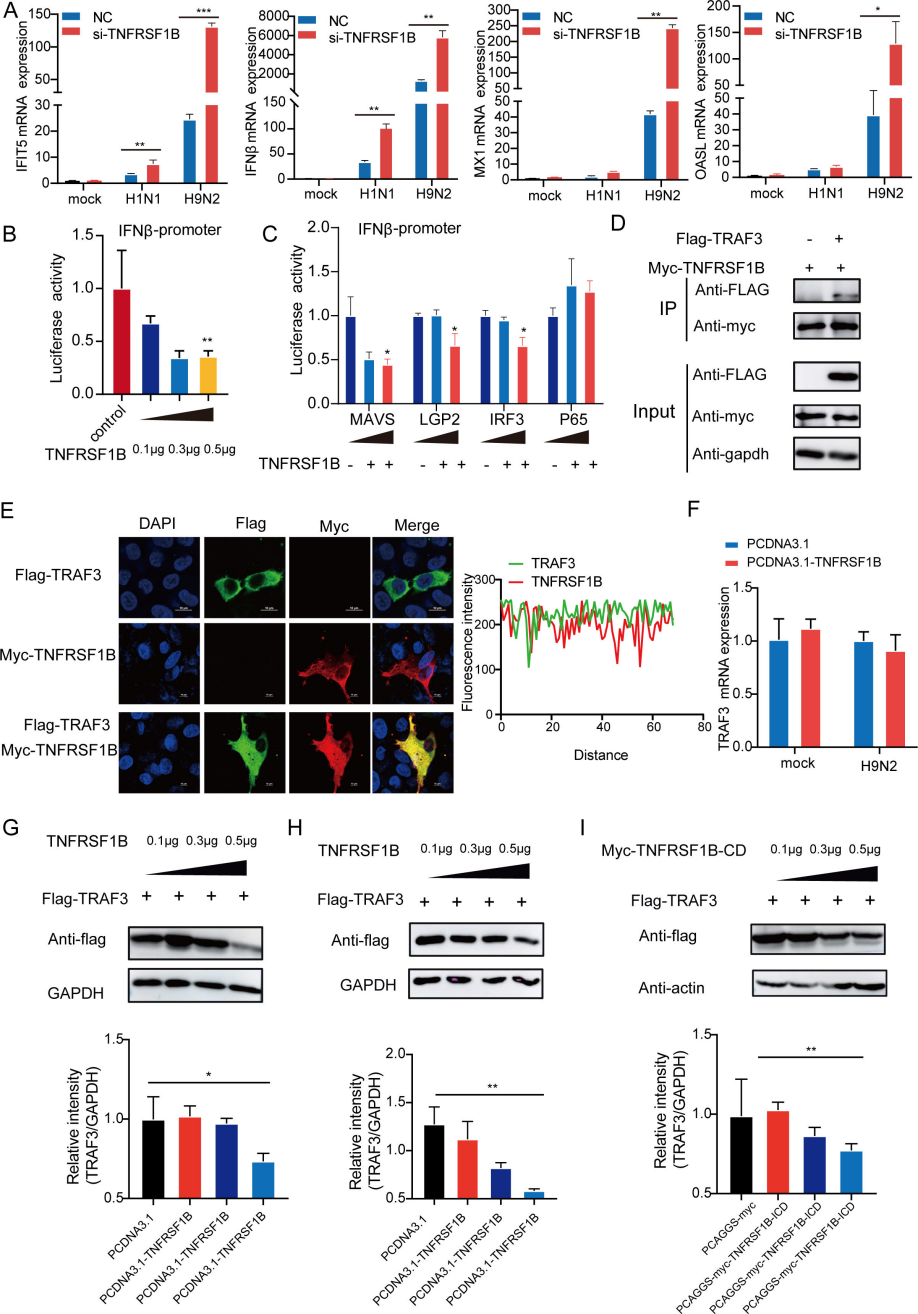
TNFRSF1B mediates the degradation of TRAF3. (**A**) DF1 cells were transfected with TNFRSF1B siRNA; at 24 h post-transfection, cells were infected with H9N2 or H1N1 influenza virus. The expression of type I interferons and interferon-stimulated genes was evaluated by q-PCR. (**B**) DF1 cells were co-transfected with the TNFRSF1B-expressing plasmid and the IFNβ promoter reporter plasmid. At 24 h post-transfection, IFNβ promoter activity was analyzed via dual-luciferase reporter assay. (**C**) DF1 cells were co-transfected with TNFRSF1B-expressing plasmid, IFNβ promoter reporter plasmid, and eukaryotic expression plasmids encoding P65, MAVS, LGP2, or IRF3, respectively. IFNβ promoter activity was assessed using a dual-luciferase reporter assay. (**D**) DF1 cells were co-transfected with Myc-tagged TNFRSF1B plasmid and Flag-tagged TRAF3 plasmid. The interaction between TNFRSF1B and TRAF3 was detected by co-immunoprecipitation (co-IP) assay. For immunoprecipitation (IP), 1 µg of anti-Myc antibody (AE010, Abclone) was used. Immunoblotting was performed with anti-Flag antibody (AE092, Abclone) and anti-Myc antibody (AE010, Abclone). (**E**) DF1 cells were co-transfected with the FLAG-tagged TNFRSF1B-plasmid and myc-tagged TRAF3 plasmid. The co-localization of TNFRSF1B and TRAF3 was examined via indirect immunofluorescence assay. Confocal fluorescence signals were quantified using ImageJ software. (**F**) DF1 cells were transfected with TNFRSF1B plasmid. At 24 h post-transfection, cells were infected with the H9N2 subtype influenza virus, and TRAF3 mRNA expression levels were determined by q-PCR at 24 h post-infection. (**G and H**) 293T or DF1 cells were co-transfected with TNFRSF1B-PCDNA3.1 plasmid and FLAG-tagged TRAF3 plasmid. TRAF3 protein levels were detected by western blotting. Quantification of three independent Western blot replicates is presented in Fig. S4A and B. (**I**) 293T cells were co-transfected with myc-tagged TNFRSF1B-ICD plasmid and Flag-tagged TRAF3 plasmid. TRAF3 protein expression levels were analyzed via western blotting. Quantification of three independent western blot replicates is presented in Fig. S4C. Data shown are the mean ± SD from three independent experiments. Western blot results are representative of three independent experiments. All statistical analyses were performed using an unpaired two-tailed *t*-test or one-way ANOVA. *, *P* < 0.05, **, *P* < 0.01, ***, *P* < 0.001.

The most well-characterized mechanism underlying miRNA-mediated gene regulation involves sequence-specific binding to the 3′ untranslated region (3′UTR) of target mRNAs. To identify potential target genes of miR-92, we first performed functional screening using DF1 cells transfected with miR-92 mimics (80 nm). At 36 h post-transfection, total RNA was extracted, and transcriptome analysis was performed using next-generation sequencing (NGS) (Fig. 5A). The efficiency of miR-92 overexpression was validated by qPCR (Fig. 5B). Transcriptome analysis revealed 256 differentially expressed genes, among which 82 genes were significantly upregulated, and 174 were downregulated (Fig. 5C). Since miRNAs typically inhibit the expression of target mRNAs, we validated the downregulated genes in the transcriptome data. Consistent with the NGS data, transfection of DF1 cells with miR-92 mimics for 36 h resulted in a significant reduction in mRNA levels of all 10 candidates (*P* < 0.05), confirming the reliability of our transcriptome profiling (Fig. 5D and E ). To further verify direct interactions between miR-92 and its candidate targets, we constructed luciferase reporter plasmids containing the wild-type 3′ UTR sequences of 10 prioritized genes. 293T cells were co-transfected with miR-92 mimics or NC alongside each reporter plasmid. Luciferase activity was measured 36 h post-transfection using the Dual-Luciferase Reporter Assay System. Among the 10 constructs, miR-92 significantly suppressed luciferase activity of the pmirGLO-TNFRSF1B-3′ UTR reporter, indicating TNFRSF1B as a strong candidate target (Fig. 5F). Of the predicted candidate targets, only TNFRSF1B exhibited consistent downregulation at both the mRNA level and in 3′ UTR luciferase activity following miR-92 overexpression. Accordingly, TNFRSF1B was identified as a candidate target gene of miR-92. Bioinformatic analysis using miRDB predicted a seven-nucleotide seed region match between miR-92 and positions 55–61 of the TNFRSF1B 3′ UTR (Fig. 5G). To confirm this interaction, we generated a mutant reporter plasmid (pmirGLO-TNFRSF1B-3′ UTR-Mut) harboring a 7 bp mutation within the predicted binding site. Co-transfection assays revealed that miR-92 suppressed luciferase activity of the TNFRSF1B-3′ UTR wild-type reporter plasmid. This inhibitory effect was completely abrogated in the mutant construct, confirming the direct and specific interaction between miR-92 and the TNFRSF1B 3′ UTR (Fig. 5H). Finally, to validate the regulatory effect of miR-92 on TNFRSF1B at the protein level, we co-transfected 293T cells with miR-92 mimics and a PCMV-Flag-TNFRSF1B-UTR expression plasmid. Western blot analysis demonstrated that miR-92 significantly reduced TNFRSF1B protein levels (Fig. 5I). This inhibitory effect was abrogated when the miR-92 binding site was mutated, confirming the specific interaction between miR-92 and the TNFRSF1B 3′ UTR (Fig. 5J). Collectively, these results provide compelling evidence that miR-92 directly targets and represses TNFRSF1B through specific binding to its 3′ UTR. To elucidate the functional role of TNFRSF1B in influenza replication, we performed siRNA-mediated knockdown experiments. DF1 cells were transfected with TNFRSF1B-specific siRNA. Following viral challenge, virus titers were determined using plaque assay, which demonstrated that TNFRSF1B knockdown significantly suppressed viral replication (Fig. 5K and L). We next examined whether transfection of miR-92 mimics or TNFRSF1B siRNA affects influenza virus entry into host cells. As shown in Fig. S1E, neither miR-92 overexpression nor TNFRSF1B knockdown exerted a significant effect on viral entry efficiency. These results indicate that the alterations in viral titers observed in our study are primarily mediated by the regulation of post-entry stages of the viral life cycle, rather than the viral entry process itself. To further delineate the mechanism underlying TNFRSF1B-mediated regulation of influenza virus replication, we investigated whether TNFRSF1B modulates type I interferon signaling. We quantified the mRNA expression of IFN-β and canonical interferon-stimulated genes (ISGs) in cells transfected with TNFRSF1B siRNA followed by influenza virus challenge. Q-PCR analysis showed that TNFRSF1B knockdown led to a significant upregulation of IFN-β and ISGs compared to control-transfected cells (Fig. 6A). Collectively, these findings suggest that TNFRSF1B functions as a negative regulator of the type I interferon signaling pathway during influenza infection.

### TNFRSF1B mediates the degradation of TRAF3 VIA the autophagolysosomal pathway

**Fig 7:**
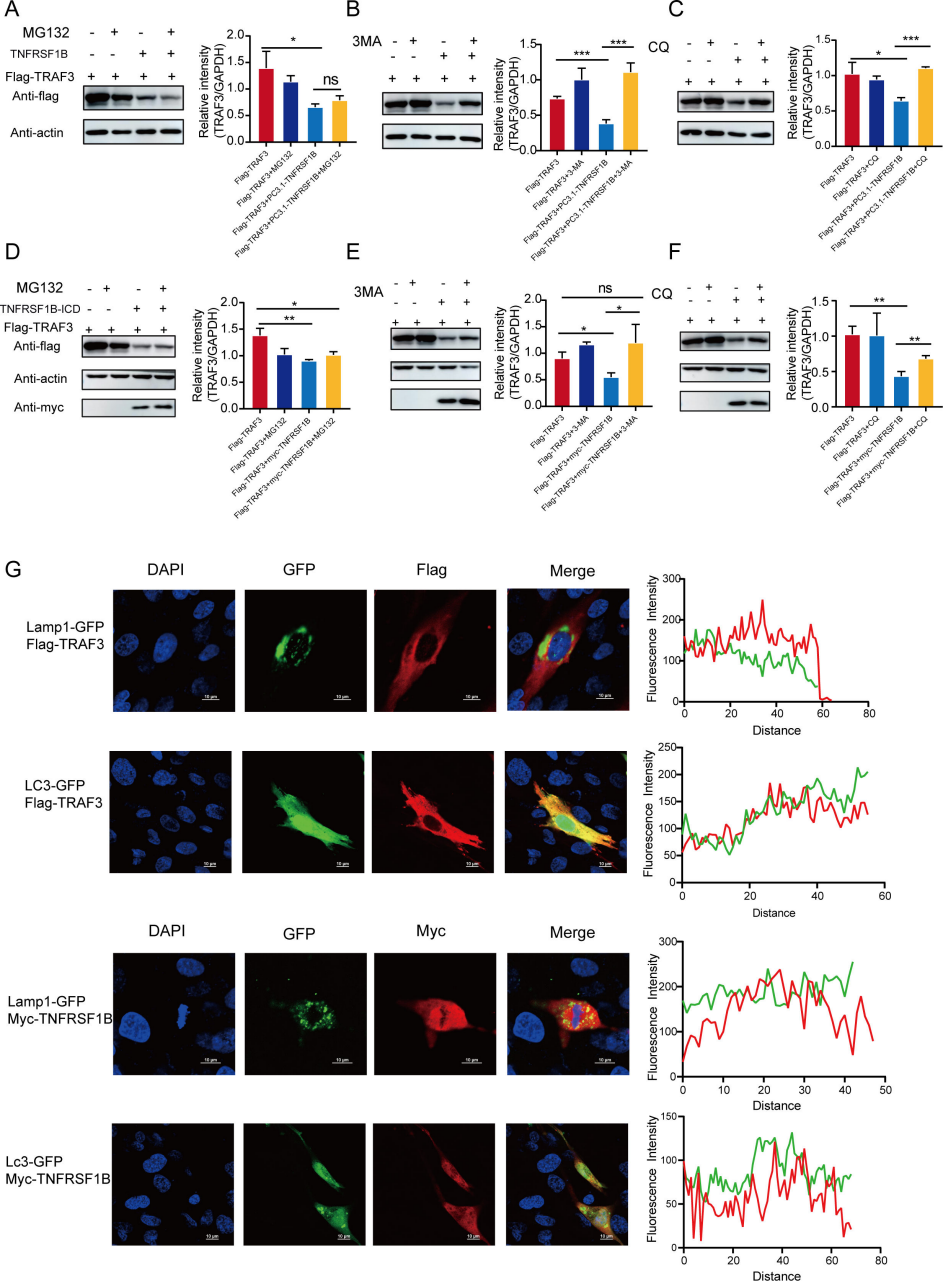
TNFRSF1B degrades TRAF3 via the autophagic pathway. (**A–C**) 293T cells were co-transfected with TNFRSF1B-PCDNA3.1 plasmid and Flag-tagged TRAF3 plasmid. At 18 h post-transfection, cells were treated with MG132 (10 µM), chloroquine (CQ, 50 µM), or 3-methyladenine (3-MA, 5 µM) for 6 h, respectively. TRAF3 protein expression was detected by western blotting. Immunoblotting was performed with anti-Flag (AE092, Abclone) and anti-GAPDH (60004-1, Proteintech) antibodies. (**D–F**) 293T cells were co-transfected with MYC-tagged TNFRSF1B-ICD plasmid and Flag-tagged TRAF3 plasmid. At 18 h post-transfection, cells were treated with MG132 (10 µM), CQ (50 µM), or 3-MA (5 µM) for 6 h, respectively. TRAF3 protein expression was analyzed via WB. Immunoblotting was performed with anti-Flag (AE092, Abclone) and anti-GAPDH (60004-1, Proteintech) antibodies. (**G**) DF-1 cells were co-transfected with MYC-tagged TNFRSF1B-ICD together with GFP-tagged LAMP1 or FLAG-tagged TRAF3 together with GFP-tagged LAMP1. The co-localization of the indicated proteins was examined by indirect immunofluorescence staining. DF-1 cells were co-transfected with MYC-tagged TNFRSF1B-ICD and GFP-tagged LC3 or FLAG-tagged TRAF3 and GFP-tagged LC3. The co-localization of each protein pair was analyzed by immunofluorescence assay. Additional fields of view are shown in Fig. S4D and E. Data shown are the mean ± SD from three independent experiments. Western blot results are representative of three independent experiments. The gray values of immunoblot bands were quantified for statistical analysis of relative protein abundance. Confocal fluorescence signals were quantified using ImageJ software. All statistical analyses were performed using unpaired two-tailed *t*-tests or one-way ANOVA. *, *P* < 0.05, **, *P* < 0.01,***, *P* < 0.001.

To further dissect the regulatory role of TNFRSF1B in the IFN signaling pathway, we performed an interferon promoter activity assay using a dual-luciferase reporter system. DF1 cells were co-transfected with a TNFRSF1B expression plasmid and an IFN-β luciferase reporter plasmid, along with a Renilla luciferase plasmid as an internal control for normalizing transfection efficiency. Luciferase activity was measured at 48 h post-transfection. The results demonstrated that overexpression of exogenous TNFRSF1B significantly suppressed IFN-β promoter activity in a dose-dependent manner (Fig. 6B). To elucidate the molecular basis underlying this inhibitory effect, we next examined whether TNFRSF1B modulates IFN-β promoter activity through key signaling intermediates. DF1 cells were co-transfected with the IFN-β luciferase reporter plasmid, TNFRSF1B expression plasmid, and individual expression plasmids encoding MAVS, LGP2, IRF3, or p65. Luciferase assays revealed that TNFRSF1B significantly attenuated IFN-β promoter activation mediated by MAVS, LGP2, and IRF3, whereas no significant inhibitory effect on p65-mediated activation was observed (Fig. 6C). Given that TNFRSF1B recruits TRAF protein family members to mediate signal transduction, we first investigated the interaction between TNFRSF1B and TRAF3. DF1 cells were co-transfected with myc-tagged TNFRSF1B and pCMV-Flag-TRAF3. At 36 h post-transfection, cell lysates were immunoprecipitated and analyzed by western blotting. Co-IP results showed a specific interaction between TNFRSF1B and TRAF3 (Fig. 6D). Confocal microscopy further confirmed this interaction, as TNFRSF1B and TRAF3 exhibited significant co-localization in DF1 cells (Fig. 6E). These findings collectively demonstrate a specific interaction between TNFRSF1B and TRAF3. TRAF3 is a conserved adaptor protein with critical roles in innate immune signaling, lymphocyte development, and cell death regulation. We next explored whether TNFRSF1B regulates TRAF3 protein expression. Quantitative RT-PCR analysis showed no significant change in TRAF3 mRNA levels upon TNFRSF1B overexpression (Fig. 6F). To assess post-translational effects, HEK293T cells were co-transfected with Flag-TRAF3 and increasing doses of pcDNA3.1-TNFRSF1B. Western blot analysis revealed a dose-dependent decrease in TRAF3 protein levels, which was recapitulated in DF1 cells, indicating TNFRSF1B promotes TRAF3 degradation (Fig. 6G and H). Given that the intracellular domain of TNFRSF1B is critical for downstream signal transduction, we generated a myc-tagged TNFRSF1B intracellular domain construct (PCAGGS-myc-TNFRSF1B-ICD, amino acids 260–462). Transfection of this truncation also reduced TRAF3 protein levels, confirming the intracellular domain is sufficient for this activity (Fig. 6I). To identify the degradation pathway involved, we treated transfected cells with MG132 (10 µM, proteasome inhibitor), chloroquine (CQ, 50 µM, lysosome acidification inhibitor), or 3-methyladenine (3MA, 10 mM, class III PI3K inhibitor). While MG132 had no effect, both CQ and 3MA restored TRAF3 protein levels in the presence of TNFRSF1B or myc-TNFRSF1B-ICD (Fig. 7A through F). These results suggest that TNFRSF1B degrades TRAF3 via the autophagolysosomal pathway. Confocal microscopy further showed co-localization of TNFRSF1B-myc and TRAF3-Flag with autophagosome marker LC3B and lysosomal marker LAMP1 (Fig. 7G). Additionally, TNFRSF1B transfection induced GFP-LC3 puncta formation, confirming the induction of autophagosome biogenesis. In parallel, western blot analysis verified that TNFRSF1B upregulates endogenous LC3 protein expression (Fig. S1F). Together, these data demonstrate that TNFRSF1B promotes TRAF3 degradation through the activation of autophagic flux.

### TNFRSF1B attenuates K63-linked ubiquitination of TRAF3

**Fig 8:**
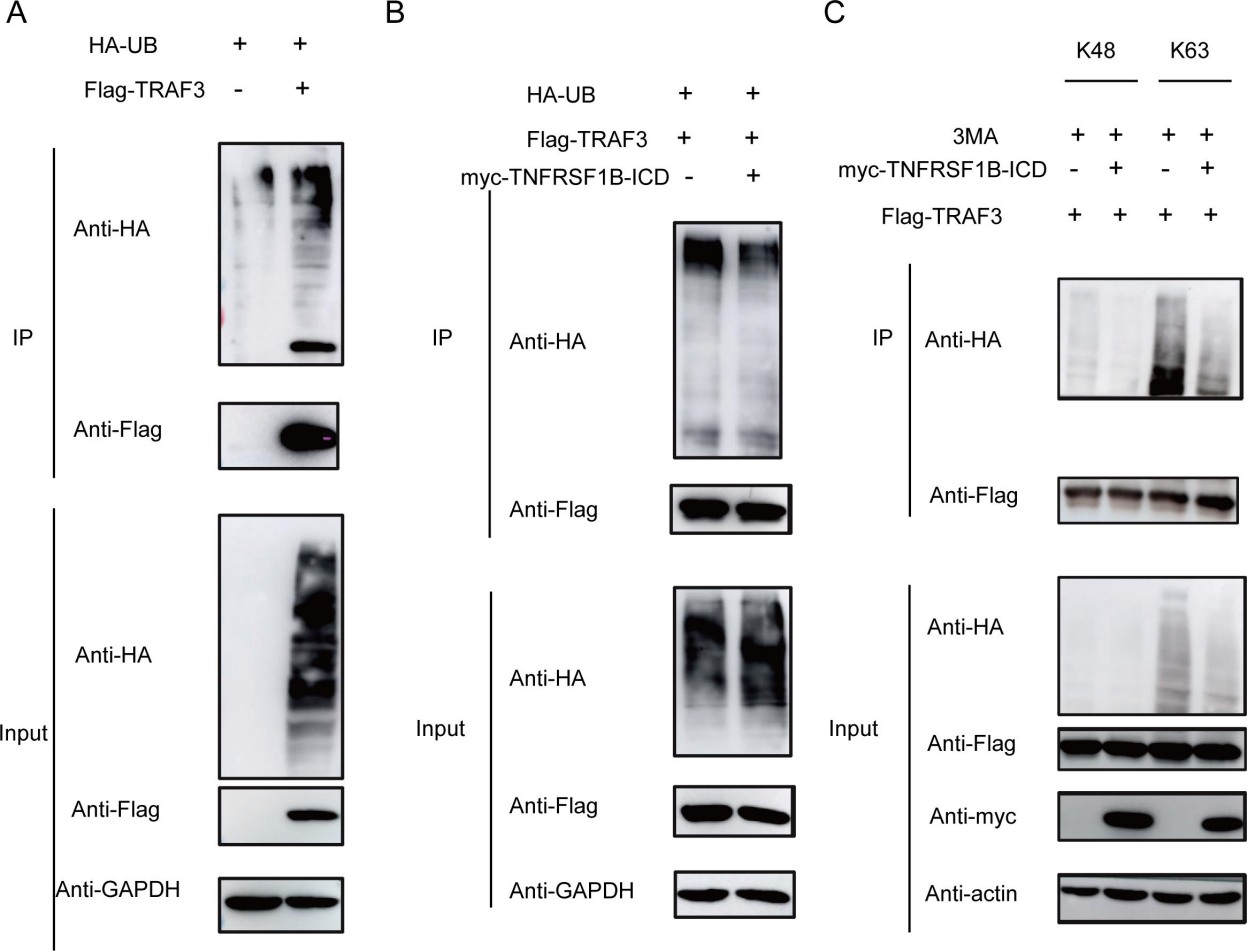
TNFRSF1B impairs K63-linked ubiquitination of TRAF3. (**A**) 293T cells were co-transfected with the FLAG-tagged TRAF3 plasmid and HA-tagged ubiquitin (HA-UB) plasmid. Ubiquitination of TRAF3 was detected by co-immunoprecipitation (co-IP) assay. For immunoprecipitation, 1 µg of anti-Flag (AE092, Abclone) was used. Immunoblotting was performed with anti-Flag (AE092, Abclone) and anti-HA (M20003, Abmart) antibodies. (**B**) 293T cells were co-transfected with MYC-tagged TNFRSF1B-ICD plasmid, FLAG-tagged TRAF3 plasmid, and HA-UB plasmid. Ubiquitination of TRAF3 was assessed via co-IP assay. (**C**) 293T cells were co-transfected with MYC-tagged TNFRSF1B-ICD plasmid, FLAG-tagged TRAF3 plasmid, and either HA-UB-K48 (K48-linked ubiquitin) or HA-UB-K63 (K63-linked ubiquitin) plasmid. Ubiquitination of TRAF3 was analyzed by co-IP assay. For immunoprecipitation (IP), 1 µg of anti-Flag antibody (AE092, Abclone) was used. Immunoblotting was performed using anti-Flag (AE092, Abclone), anti-Myc (AE010, Abclone), and anti-HA (M20003, Abmart) antibodies.

Ubiquitination of TRAF3 represents a key post-translational modification that governs the activation of the type I interferon pathway. Specifically, K63-linked polyubiquitination of TRAF3 has been identified as a critical event in innate immune signaling, as it promotes the formation of signaling complexes required for downstream antiviral responses. Previous studies have demonstrated that TRIM24 directly interacts with TRAF3 and catalyzes its K63-linked ubiquitination (24). This modification stabilizes TRAF3 and facilitates its association with MAVS and TBK1, thereby triggering the phosphorylation and nuclear translocation of IRF3 to induce IFN production. Based on the above background, we explored the functional role of TRAF3 during influenza virus infection. DF1 cells were transfected with TRAF3 siRNA prior to influenza virus challenge. Western blot and qPCR results revealed that TRAF3 knockdown markedly suppressed the expression of interferons and interferon-stimulated genes (Fig. S2A and B). We next investigated whether miR-92 modulates TRAF3 protein expression through TNFRSF1B. Individual knockdown of TNFRSF1B or transfection of miR-92 mimics elevated endogenous TRAF3 protein levels, confirming that TNFRSF1B acts as an upstream negative regulator of TRAF3 (Fig. S2C). In rescue experiments, combined transfection of miR-92 mimics and TNFRSF1B siRNA partially reversed the inhibitory effect of miR-92 on TRAF3 expression. The partial rescue phenotype indicated that gga-miR-92 modulates TRAF3 abundance predominantly via the TNFRSF1B signaling axis, while additional auxiliary target genes may also synergistically participate in this regulatory network. We then further confirmed the ubiquitination of avian TRAF3. 293T cells were co-transfected with FLAG-TRAF3 and the HA-tagged ubiquitin expression plasmid (HA-Ub) to monitor ubiquitination events. At 24 h post-transfection, cells were lysed, and immunoprecipitation was performed. Western blot analysis with anti-HA antibody revealed that TRAF3 is indeed subject to ubiquitination (Fig. 8A). To investigate the impact of TNFRSF1B on TRAF3 ubiquitination, we performed co-transfection experiments. Co-immunoprecipitation assays demonstrated that TNFRSF1B overexpression significantly reduced the ubiquitination level of TRAF3 (Fig. 8B). To determine the specific ubiquitin linkage affected, we repeated the experiments using HA-tagged K63-ubiquitin (HA-K63-Ub), which contains a lysine residue only at position 63. Notably, TNFRSF1B overexpression led to a marked reduction in K63-linked ubiquitination of TRAF3(Fig. 8C). These results indicate that TNFRSF1B specifically antagonizes the K63-linked ubiquitination of TRAF3, thereby potentially impairing its ability to activate the IFN-I signaling pathway.

## DISCUSSION

miRNA-mediated gene silencing has emerged as a fundamental mechanism underlying host defense against viral infections. Previous studies have demonstrated the feasibility of miRNA-based viral attenuation strategies. For instance, an influenza virus engineered to harbor miR-192 target sequences exhibited significantly attenuated pathogenicity in murine models, with reduced viral titers in respiratory tissues (25). Similarly, rational modification of influenza viruses to incorporate miR-21 target sequences led to consistent attenuation phenotypes across diverse hosts, thereby highlighting their potential as live attenuated vaccine candidates (26). These findings underscore the importance of miRNA-based attenuation approaches. In this study, we present an avian-origin miRNA that exhibits the ability to suppress influenza virus replication. This discovery offers valuable insights into the development of antiviral strategies utilizing avian miRNAs. We observed that miR-92 suppressed H9N2 avian influenza virus replication in DF1 cells but exerted no significant inhibitory effect in A549 cells. This discrepancy may arise from multiple factors. Although human and avian miR-92 are highly conserved, their corresponding target mRNAs differ substantially between human and avian cells and lack evolutionary conservation. As a result, miR-92 may fail to regulate its target mRNAs in human cells, thereby being unable to restrict H9N2 replication in A549 cells. Notably, in A549 cells, miR-92 inhibited the replication of H1N1 influenza virus but not H9N2 avian influenza virus, which may be attributed to viral subtype-specific features. Avian influenza viruses generally exhibit limited replication efficiency in mammalian cells (27, 28). Accordingly, infection of A549 cells with avian and human influenza viruses triggers distinct host gene expression patterns. The differential expression of specific host genes induced by H9N2 infection in A549 cells may consequently attenuate the regulatory function of miR-92.

**Fig 9:**
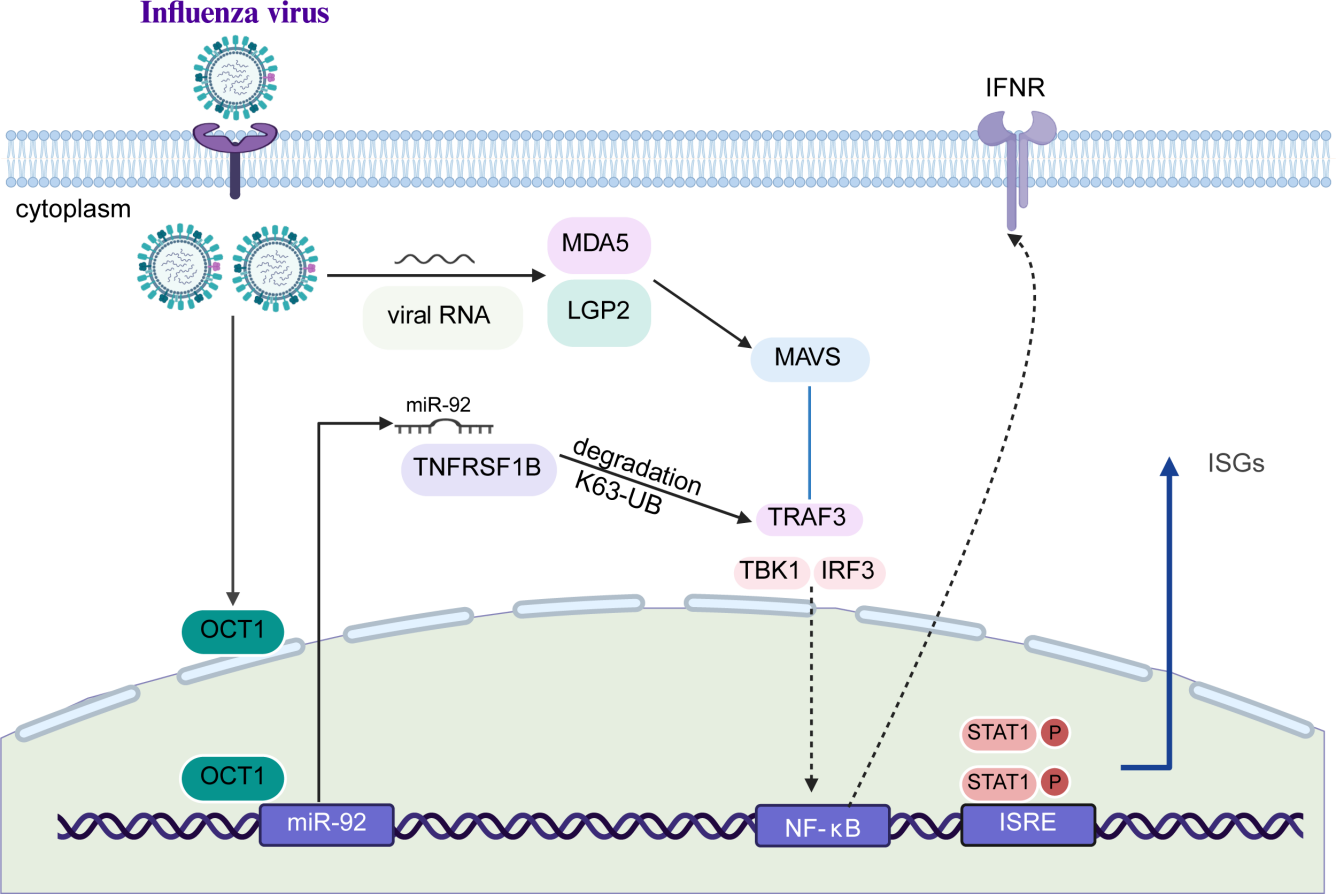
Schematic of miR-92 transcription and its regulation of influenza virus replication. Influenza virus activates the transcription of miR-92 via the transcription factor OCT1. In turn, miR-92 targets TNFRSF1B, which not only inhibits influenza virus replication but also promotes the production of type I interferons. Mechanistically, TNFRSF1B mediates the degradation of TNF receptor-associated factor 3 (TRAF3) through the autophagolysosomal pathway, thereby suppressing interferon signaling.

The miR-17-92 gene cluster, localized on chromosome 1, encodes a set of six mature miRNAs, including miR-17, miR-18a, miR-19a, miR-20a, miR-19b, and miR-92 (29). This cluster has emerged as a critical player in antiviral defense, with multiple members exhibiting distinct inhibitory effects against viral pathogens. For example, miR-18a-5p exerts its antiviral activity against the influenza virus by directly targeting the NEDD9 gene, thereby disrupting viral replication (30). MiR-19a and miR-19b have been shown to suppress influenza virus replication in A549 cells through specific targeting of SOCS1, a key regulator of host immune responses (31). In the present study, we extend these findings by identifying another avian-origin miRNA within this cluster, miR-92, as a potent inhibitor of influenza virus replication, mediated through its direct targeting of TNFRSF1B (Fig. 9). The expression of miRNA is tightly regulated by transcription factors, and miRNA clusters often share conserved regulatory regions to ensure coordinated expression (32, 33). Previous studies have demonstrated that members of the E2f family can bind to the promoter region of the human miR-17-92 cluster, thereby activating its transcription (34). In our investigation, we identified a GC-rich region upstream of the avian miR-92 gene that exhibits robust promoter activity. Functional assays revealed that specific avian transcription factors enhance the promoter activity of the miR-92 gene, consequently promoting its transcription. Conversely, knockdown of the transcription factor OCT1 resulted in reduced promoter activity of miR-92 and suppressed the transcription of miR-92. Furthermore, chromatin immunoprecipitation assays confirmed that the transcription factor OCT1 binds directly to the promoter region of the avian miR-17-92 cluster. These results strongly suggest that OCT1 acts as a key regulator of miR-92 transcription. Notably, sequence homology analysis of the promoter regions between avian and human miR-17-92 clusters suggests that these regulatory mechanisms are evolutionarily conserved, providing a cross-species framework for understanding miRNA cluster regulation.

TNFRSF1B, a key immunomodulatory receptor belonging to the TNF receptor superfamily (TNFRSF), exerts its biological functions through intricate interactions with TNF receptor-associated factor (TRAF) family members (35). In our study, we found that knockout of TNFRSF1B can inhibit influenza virus replication and promote the expression of type I interferons. Subsequent mechanistic investigations revealed that TNFRSF1B can directly suppress the transcriptional activity of interferon promoters, establishing a functional link between TNFRSF1B signaling and the host antiviral IFN response. Previous studies have demonstrated that mammalian TNFRSF1B recruits the cellular inhibitor of apoptosis protein 1 (cIAP1) to mediate TRAF2 degradation via the ubiquitin-proteasome pathway, thereby dampening TNFR1-dependent NF-κB activation (36). Intriguingly, our findings reveal a distinct regulatory mechanism. Avian TNFRSF1B primarily targets TRAF3 for degradation, and this process is mediated through the autophagosome-lysosome pathway rather than the ubiquitin-proteasome system. Further domain mapping experiments identified the cytoplasmic domain of avian TNFRSF1B as the critical determinant for TRAF3 degradation. The functional significance of TRAF3 degradation by avian TNFRSF1B is underscored by TRAF3’s well-established role as a pivotal signal transducer in antiviral immunity. TRAF3 is known to positively regulate the interferon response, thereby restricting influenza virus replication (37). Our observations support the hypothesis that TNFRSF1B-mediated TRAF3 degradation represents a key mechanism by which TNFRSF1B inhibits the interferon signaling pathway.

TRAF3 contains an N-terminal RING finger domain with E3 ubiquitin ligase activity, five zinc finger motifs, and a C-terminal TRAF domain that mediates receptor binding and homo-oligomerization (38). TRAF3 functions as a central hub in multiple antiviral signaling cascades. For instance, TRAF3 is essential for TLR-mediated type I IFN production, as demonstrated by the impaired IFN response in TRAF3-deficient macrophages and dendritic cells (39). During viral infection, cytosolic double-stranded RNA is recognized by the RIG-I-like receptor (RLR) family member RIG-I, triggering a conformational change that exposes its CARD domain (40). This activated RIG-I then interacts with the CARD domain of the adaptor protein MAVS, promoting MAVS oligomerization (41). The oligomerized MAVS platform subsequently recruits TRAF3, which, in turn, assembles the IKKε/TBK1 kinase complex to phosphorylate and activate IRF3, ultimately initiating interferon gene transcription (42, 43). Building on these findings, our study identifies an additional mechanism by which avian TNFRSF1B modulates TRAF3-dependent signaling. We observed that TNFRSF1B enhances the removal of K63-linked ubiquitin chains from TRAF3. Given that K63-linked ubiquitination is a critical post-translational modification for TRAF3-mediated signal transduction (44), its removal by TNFRSF1B impairs TRAF3’s signaling function. This deubiquitinating activity of TNFRSF1B likely disrupts the structural integrity of TRAF3 signaling complexes. Collectively, these findings reveal a multi-layered inhibitory strategy employed by avian TNFRSF1B to dampen interferon signaling, which may serve as a conserved host regulatory mechanism.

In summary, we identified miR-92 as an interferon-related miRNA that facilitates interferon responses by targeting TNFRSF1B. Importantly, we found that the activation of the transcription factor OCT1 promotes the expression of miR-92. These data provide mechanistic insight into how miRNAs affect influenza virus survival and immune escape.

## MATERIALS AND METHODS

### Cells and viruses

HEK-293T (human embryonic kidney 293T), MDCK (Madin-Darby canine kidney), and DF-1 (chicken embryo fibroblast 1) cells were maintained in Dulbecco’s modified Eagle medium (DMEM, Gibco, USA) supplemented with 10% fetal bovine serum and 1% penicillin-streptomycin (Beyotime, Shanghai, China). A/chicken/Anhui/LH99/2017(H9N2) and A/WSN/1933(H1N1) viral strains were propagated in SPF chicken embryos, aliquoted, and stored at −80°C until use. All experiments with infectious viruses were conducted in a Biosafety Level 2 (BSL-2) laboratory.

### miRNA sequencing

DF-1 cells (a chicken embryo fibroblast cell line widely used for avian influenza virus research) were infected with the H9N2 avian influenza virus at a multiplicity of infection (MOI) of 3, and cell samples were harvested at 12 hours post-infection (hpi). Total RNA was extracted using TRIzol reagent (Vazyme, Nanjing, China). Subsequently, RNA molecules with a size of 18-30 nt were enriched through polyacrylamide gel electrophoresis (PAGE) fractionation. The resulting cDNA library was sequenced on an Illumina HiSeq X Ten platform. Raw sequencing reads were subjected to quality filtering to remove low-quality reads, adapter sequences, and other contaminants. Clean reads were then aligned to the reference genome. Differential expression analysis of miRNAs between infected and uninfected groups was performed using edgeR software (45), with significantly differentially expressed miRNAs defined as those with a fold change > 2 and a *P*-value < 0.05.

### miRNA mimics

The miRNA mimics were synthesized by GenePharma (Shanghai, China). The sequences are shown in Table 1.

**TABLE 1:** miRNA mimic sequences used in this study

miRNA mimic	Sense sequence (5′−3′)	Anti-sense sequence (5′−3′)
miR-92 mimics	AGGUUGGGAUCAGUUGCAAUGCU	CAUUGCAACUGAUCCCAACCUUU
miR-197-3p mimics	UUCACCACCUUCUCCACCCAGC	UGGGUGGAGAAGGUGGUGAAUU
miR-1237-3p mimics	UCCUUCUGCUCCGUCCCCCAG	GGGGGACGGAGCAGAAGGA UU
miR-1709 mimics	UGGAAUGAUGAGUGCACUGACU	UCAGUGCACUCAUCAUUCCAUU
miR-375 mimics	UUUGUUCGUUCGGCUCGCGUUA	ACGCGAGCCGAACGAACAAA UU
miR-122-5p mimics	UGGAGUGUGACAAUGGUGUUUGU	AAACACCAUUGUCACACUCCAUU
miR-215 mimics	AUGACCUAUGAAUUGACAGAC	CUGUCAAUUCAUAGGUCAUUU
miR-204 mimics	UUCCCUUUGUCAUCCUAUGCCU	GCAUAGGAUGACAAAGGGAAUU
miR-34b mimics	CAGGCAGUGUAGUUAGCUGAUUG	AUCAGCUAACUACACUGCCUGUU
miR-141-3p mimics	UAACACUGUCUGGUAAAGAUGG	AUCUUUACCAGACAGUGUUAUU

### Construction of plasmids

To verify the activity of the miR-92 promoter, the promoter region of miR-92 was amplified by PCR using specific primers containing XhoI and BgIII restriction enzyme sites. The amplified fragment was subcloned into the pGL3-basic firefly luciferase reporter vector. To validate the direct targets of miR-92, the full-length 3′-UTR of candidate target genes containing the miR-92 binding site was amplified by PCR with primers harboring XhoI and SaII restriction sites. The PCR product was inserted into the pmirGLO dual-luciferase reporter vector. The IFNβ promoter luciferase reporter and the PRL-TK internal control plasmids have been previously constructed and preserved in our laboratory. To identify transcription factors regulating miR-92 expression, the coding sequence (CDS) of candidate transcription factors was amplified by PCR with primers containing EcoRI and XhoI restriction sites and cloned into the pcDNA3.1 expression vector. For OCT1 overexpression analysis, the CDS of OCT1 was amplified using primers with HindIII and XhoI restriction sites, and inserted into the pCMV-FLAG vector (treated with HindIII and XbaI). For TRAF3 protein degradation assays, the CDS of TRAF3 was amplified by PCR with primers harboring HindIII and XhoI restriction sites and cloned into both pCMV-FLAG and pCAGGS-MYC expression vectors. The intracellular domain and UTR region of TNFRSF1B were amplified using primers containing HindIII and XhoI restriction sites and subcloned into the pCMV-FLAG vector. All constructed plasmids were verified by DNA sequencing to ensure sequence accuracy. All plasmids were constructed using a homologous recombination kit (Cat No. C112, Vazyme). The sequences of the cloning primers are listed in Table 2.

**TABLE 2:** Primers used for plasmid cloning in this study

Primer	Sequence (5′−3′)
CEPCMV-TRAF3-F	aaggacgacgatgacaagcttATGGACACCAGTAAGAAGACAGAACC
CEPCMV-TRAF3-R	agatctcggtcgaccgaattcTCAGGGGTCTGGTAGATCCGA
PCAGGSmyc--TNFRSF1B-ICD-F	tcagaagaggatctggaattcTCCAAAAAAAAAGCCCTTGCC
PCAGGSmyc--TNFRSF1B-ICD-R	attaagatctgctagctcgagTTAAACAGTTTTCATCCCCATATCTT
CEPCMV-OCT1-F	aaggacgacgatgacaagcttATGAACAATCCGTCAGAAACCAG
CEPCMV-OCT1-R	agatctcggtcgaccgaattcTCACTGTGCCTTGGAGGCA
CEPCMV-mavs-F	aaggacgacgatgacaagcttATGGGTTTCGCCGAGGAC
CEPCMV-mavs-R	agatctcggtcgaccgaattcCTATTTCTGCAATCGTGTGTACACC
PCDNA3.1-CEBPA-F	tagtccagtgtggtggaattcATGGAGCAAGCCAACTTCTACG
PCDNA3.1-CEBPA-R	aacgggccctctagactcgagCTAGGCGCAGCTGCCCAT
PCDNA3.1-AR-F	tagtccagtgtggtggaattcATGGAGGTGCAGCTGGGG
PCDNA3.1-AR-R	aacgggccctctagactcgagTCACTCCGCGTGGAAGTAAATG
PCDNA3.1-MAFF-F	tagtccagtgtggtggaattcATGGCTGCGGATGGGCTG
PCDNA3.1-MAFF-R	aacgggccctctagactcgagCTAGGAGTAGGCGGCCTGGT
PCDNA3.1-IRF1-F	tagtccagtgtggtggaattcATGCCCGTCTCAAGGATGC
PCDNA3.1-IRF1-R	aacgggccctctagactcgagTTACAAGCTGCAGGAGATGGC
PCDNA3.1-jun-F	tagtccagtgtggtggaattcATGAGTGCAAAGATGGAGCCTAC
PCDNA3.1-jun-R	aacgggccctctagactcgagTCAAAACGTTTGCAACTGTTGTG
PCDNA3.1-fos-F	tagtccagtgtggtggaattcATGATGTACCAGGGCTTCGCT
PCDNA3.1-fos-R	aacgggccctctagactcgagTCACAAGGCCAGCAGGGTG
PCDNA3.1-PRDM1-F	tagtccagtgtggtggaattcATGAAAATGGACATGGAGGATGC
PCDNA3.1-PRDM1-R	aacgggccctctagactcgagTTAAGGGTCCATTGGTTCAACTG
ce-glo-kpna2utr-F	aacgagctcgctagcctcgagGCACTGCATACACTGCAACTACC
ce-glo-kpna2utr-R	cttgcatgcctgcaggtcgacTTTGATGTACTGTTAACACTTTATTGAACTT
ceglo-UHRF1utr-F	aacgagctcgctagcctcgagTTCCATAAAGCACTTCTAATTTTCTTTT
ceglo-UHRF1utr-R	cttgcatgcctgcaggtcgacTTTCATCTGAAAAGATATTTATTTTGAAAA
ceglo-trim58utr-F	aacgagctcgctagcctcgagCAGGGACAGACAGAGCTGTGG
ceglo-trim58utr-R	cttgcatgcctgcaggtcgacTCCTCAGATCATCTCTTTATTGCCT
ceglo-RRM2utr-F	aacgagctcgctagcctcgagAAGGACTGAGGCATGAGTTAGTGA
ceglo-RRM2utr-R	cttgcatgcctgcaggtcgacTTAACACACAGCTTTGCCCATT
ceglo-NTN4utr-F	aacgagctcgctagcctcgagACACATGCGGCCACCTAGG
ceglo-NTN4utr-R	cttgcatgcctgcaggtcgacTTGAAATCATAATATATTTATTACATACATGAGA
ceglo-PRIM2utr-F	aacgagctcgctagcctcgagACAACTATGGTAGCTTTCTTCTTTTCC
ceglo-PRIM2utr-R	cttgcatgcctgcaggtcgacTTCACTTAATACCATCTTTATTGACTCAGA
ceglo-MARCH1utr-1-F	aacgagctcgctagcctcgagTGGAGACAGCATTGTTTCTTCCT
ceglo-MARCH1utr-1-R	cttgcatgcctgcaggtcgacGACTCAAACATTAATCCAACCCACC
ceglo-MCM5utr-F	aacgagctcgctagcctcgagCCCCACGTCCCCTGCCTG
ceglo-MCM5utr-R	cttgcatgcctgcaggtcgacTACCAATACCAGCTTTTATTTTAAAACA
ceglo-LPCAT2utr-F	aacgagctcgctagcctcgagGAACAATCAAAGAATTCATCTCCTGA
ceglo-LPCAT2utr-R	cttgcatgcctgcaggtcgacTCTTTTAAAAATATAGGTTTATTTTAAGCACA
ceglo-TNFRSF1Butr-F	aacgagctcgctagcctcgagAGGAAATGACAGATTTTATACTGATTGAC
ceglo-TNFRSF1Butr-R	cttgcatgcctgcaggtcgacTGGATCAATCCTCTTTTAATTAAGCA
TNFRSF1B-UTRMUT-F	ATCAGCCCTTATGTAGGGTTGGAAATGTGTTAAATGTAGCAT
TNFRSF1B-UTRMUT-R	ATGCTACATTTAACACATTTCCAACCCTACATAAGGGCTGAT
PCDNA3.1-LEF1-F	tagtccagtgtggtggaattcATGCCGCAGCTGCCGGGG
PCDNA3.1-LEF1-R	aacgggccctctagactcgagTTAACAAGCTTCCATCTCCAGCA
PCDNA3.1-NFYA-F	tagtccagtgtggtggaattcATGGAGCAGTACACAGCCAACA
PCDNA3.1-NFYA-R	aacgggccctctagactcgagTTAGGAGACTCTGATCATCTGTGTCA
PCAGGS-myc--TRAF3-F	tcagaagaggatctggaattcATGGACACCAGTAAGAAGACAGAACC
PCAGGS-myc--TRAF3-R	attaagatctgctagctcgagTCAGGGGTCTGGTAGATCCGA

### Cell transfection and viral infections

Cell transfection was performed using Lipofectamine 2000 (Invitrogen, USA) following the manufacturer’s protocol. For the virus infection assay, DF-1 cells were seeded in 24-well plates and inoculated with H9N2 influenza virus. Viruses were diluted in DMEM culture medium, and the same DMEM medium was used for mock infection as a control. After 1 h of incubation at 37℃ with 5% CO2, the inoculum was discarded, and the cells were washed twice with phosphate-buffered saline (PBS). Cells were then overlaid with DMEM supplemented with TPCK-treated trypsin (L-1-tosylamido-2-phenylethyl chloromethyl ketone) at a final concentration of 0.3 µg/ml. Following incubation at 37℃ with 5% CO2 for 24 h, both cells and supernatants were harvested for downstream analyses.

### Plaque assay

For plaque assay, MDCK cells were seeded in 24-well plates and cultured overnight to reach 90%–95% confluency. Cells were inoculated with 100 µL of 10-fold serial dilutions of viral supernatants. After 1 h of adsorption at 37℃ with 5% CO2, the inoculum was carefully removed. Cells were washed once with phosphate-buffered saline to eliminate unbound virus. Subsequently, cells were overlaid with 500 µL per well of DMEM supplemented with 2% low-melting-point agarose (Lonza) and 1 µg/mL TPCK-treated trypsin. After 48 h of incubation at 37℃ with 5% CO2, plaques were counted, and viral titers were calculated as plaque-forming units per milliliter (PFU/mL).

### RNA-seq analysis for MIR-92 targets

To investigate the regulatory effects of miR-92 on host gene expression, DF-1 cells were transfected with a miR-92 mimic (80 nM final concentration) using Lipofectamine 2000 (Invitrogen, USA) according to the manufacturer’s protocol. Cells transfected with a non-targeting negative control (NC) mimic served as the control group. At 24 h post-transfection, cells were harvested, and total RNA was extracted using TRIzol reagent (Vazyme, Nanjing, China) following the standard protocol. RNA integrity was assessed using an Agilent 2100 Bioanalyzer (Agilent Technologies, Santa Clara, CA, USA). To analyze the differential expression of genes, RNA sequencing was performed with an Illumina HiSeq 4000 instrument. Differential gene expression analysis was performed using the DESeq2 package (46). The resulting *P*-values were adjusted using the Benjamini-Hochberg approach to control the false discovery rate (FDR). The FDR < 0.05 found by DESeq2 was used as the significance threshold for the identification of differentially expressed genes.

### miRNA target prediction

To identify the potential host cellular targets of miRNAs, particularly miR-92, we performed *in silico* predictions using TargetScan (47) and miRDB (48). To investigate transcriptional regulation of miR-92, we analyzed its promoter region for potential transcription factor binding sites. Prediction analysis was performed using the AnimalTFDB database.

### RNA isolation and quantitative real-time PCR (qPCR)

Total RNA was isolated from cell samples using TRIzol reagent (Vazyme, Nanjing, China) following the manufacturer’s protocol. For mRNA quantification, 1 µg of total RNA was reverse-transcribed into cDNA using the HiScript III 1st Strand cDNA Synthesis Kit (Vazyme, Nanjing, China). QPCR was performed on the LightCycler System (Roche) using SYBR Green Supermix (Accurat Biology, AG11701) with the following cycling conditions: 95℃for 30 s, followed by 40 cycles of 95℃ for 5 s, and 60℃ for 30 s. For mRNA expression analysis, GAPDH was used as an internal reference gene for normalization. For miRNA quantification, stem-loop reverse transcription was performed using specific stem-loop RT primers. Briefly, 1 µg of total RNA was reverse-transcribed into cDNA using reverse transcriptase with the designed stem-loop primers targeting mature miR-92. QPCR was then carried out using miRNA-specific forward and reverse primers. The relative expression level of miR-92 was normalized to U6 small nuclear RNA and calculated using the 2−ΔΔCt method. All reactions were performed in triplicate.

### Dual-luciferase reporter assays

To evaluate the transcriptional activity of the miR-92 promoter, 293T cells were co-transfected with the miR-92 promoter firefly luciferase reporter plasmid and the Renilla luciferase vector (pRL-TK) using Lipofectamine 2000 (Invitrogen). The pRL-TK vector served as an internal control to normalize for transfection efficiency. For analyzing transcription factor-mediated regulation of miR-92, DF-1 cells were co-transfected with miR-92 promoter firefly luciferase reporter plasmid, transcription factor expression plasmid, and pRL-TK. In parallel, knockdown experiments were performed by co-transfecting 80 nM small-interfering RNA (siRNA) targeting the transcription factor alongside the reporter plasmids. To validate miRNA-mRNA interactions, 293T cells were co-transfected with firefly luciferase reporter plasmids containing the 3′ untranslated region (3′ UTR) of the candidate target gene (or a mutant 3′ UTR with disrupted miRNA binding sites), miR-92 mimic (or NC mimic), and pRL-TK. At 24 h post-transfection, cell extracts were prepared using lysis buffer, and luciferase activities were measured using the Dual-Luciferase Reporter Assay System (Vazyme, Nanjing, China). Relative luciferase activity was calculated as the ratio of firefly luciferase activity to Renilla luciferase activity, with each assay performed in triplicate.

### Confocal microscopy

To visualize the subcellular localization of target proteins, DF-1 cells were seeded in 35 mm confocal dishes and cultured overnight. Cells were either transfected with expression plasmids (using Lipofectamine 2000) or infected with IAV (MOI = 1) as experimental groups. At 24 h post-transfection or hours post-infection, cells were fixed with 4% paraformaldehyde for 15 min at room temperature. Cells were then permeabilized with 0.3% Triton X-100 in PBS for 10 min and blocked with 5% bovine serum albumin (Sigma-Aldrich) in PBS for 1 h to reduce non-specific antibody binding. Primary antibodies were added to the dishes and incubated overnight at 4℃. After three washes with PBS, cells were incubated with Alexa Fluor 488-conjugated or Alexa Fluor 594-conjugated secondary antibodies (Invitrogen, USA) for 1 h at room temperature. Nuclei were counterstained with DAPI (Solarbio,China) for 10 min. Images were acquired using the Nikon confocal microscope.

### Western blot

DF-1 cells or 293T cells were lysed in NP-40 lysis buffer (Beyotime, China) supplemented with 1× protease inhibitor cocktail (Beyotime, China) on ice for 30 min. The lysates were centrifuged at 12,000 RPM for 10 min at 4℃, and the supernatants were collected. Protein concentration was mixed with 5× SDS-PAGE loading buffer (Beyotime, China) and denatured by boiling at 95°C for 5 min. Proteins were separated by SDS-PAGE using 10% polyacrylamide gels and transferred onto nitrocellulose (NC) membranes (0.2 µm) via a wet transfer system (Bio-Rad). After transfer, membranes were blocked with 5% non-fat dry milk for 1 h at room temperature to block non-specific binding sites. Primary antibodies were incubated with the membranes overnight at 4℃. Membranes were then washed and incubated with horseradish peroxidase (HRP)-conjugated secondary antibodies for 1 h at room temperature. Protein bands were visualized using an enhanced chemiluminescence (ECL) detection kit (Vazyme, China) and imaged using the GE Amersham Imager 600 System.

### Co-immunoprecipitation

DF1 cells were lysed with NP-40 lysis buffer, and the lysate was centrifuged at 12,000 rpm for 10 min at 4°C; 20 µL aliquot of the lysate was collected as the input sample. The remaining lysate was incubated with the IP antibody at 4°C overnight. Subsequently, the protein-antibody complexes were mixed with Protein A/G magnetic beads (BEAVER) and incubated for 4 h. The beads were washed three times with PBS, followed by lysis in 1× loading buffer. Both the whole cell lysate (input) and the immunoprecipitated (IP) eluate were analyzed by western blot.

### Bioinformatics analyses

Pearson correlation coefficients between two parallel replicates were calculated to evaluate the repeatability and reliability of the experiments. Principal component analysis (PCA) was performed using R software (v4.0.0), with the prcomp package to visualize sample similarity. A correlation coefficient closer to 1 indicates higher reproducibility between replicates. GO functional classification and enrichment analysis of differentially expressed genes (DEGs) were carried out using the GO database (http://www.geneontology.org/). Significantly enriched GO terms, encompassing molecular function, cellular component, and biological process, were identified using the hypergeometric test against the genomic background. KEGG pathway enrichment analysis was performed using the Kyoto Encyclopedia of Genes and Genomes (KEGG) database (49) to identify significantly enriched metabolic or signal transduction pathways in DEGs. Statistical significance was determined using the hypergeometric test with FDR correction (FDR ≤ 0.05), following the same statistical framework as the GO analysis.

### Statistical analysis

Data are shown as the mean ± standard deviation (SD) of three independent experiments. Statistical analysis was performed using GraphPad Prism 8. Differences between groups were evaluated by Student’s *t*-test or one-way ANOVA, with *P* < 0.05 regarded as statistically significant.

## Data Availability

The publicly available miRNA sequencing data set analyzed in this study can be accessed through the NCBI GEO database under the accession number GSE147658. High-throughput miRNA sequencing data generated in this study are included in the supplemental material. All other raw experimental data are available from the corresponding author upon reasonable request.

## SUPPLEMENTAL MATERIAL

Data Set S1 jvi.00674-26-s0001.xls High-throughput miRNA sequencing data.

Figure S1 jvi.00674-26-s0002.tif Analysis of miRNA profiles in avian cells infected with three H9N2 influenza A virus strains.

Figure S2 jvi.00674-26-s0003.tif TRAF3 knockdown regulates interferon response upon H9N2 influenza virus infection.

Figure S3 jvi.00674-26-s0004.tif Quantitative analysis of Western blot results and subcellular localization of OCT1 upon influenza virus infection.

Figure S4 jvi.00674-26-s0005.tif Quantification of Western blot results and co-localization analysis of TNFRSF1B and TRAF3 with lysosomal and autophagy markers.

Supplemental legends jvi.00674-26-s0006.docx Legends for Fig. S1 to S4.

ASM does not own the copyrights to Supplemental Material that may be linked to, or accessed through, an article. The authors have granted ASM a non-exclusive, world-wide license to publish the Supplemental Material files. Please contact the corresponding author directly for reuse.
