# Supplementary figures and images for: Gga-miR-92-targeted TNFRSF1B inhibits the replication of influenza A virus by degrading TRAF3
Source: J Virol. 2026 Jun 12;100(7):e00674-26. doi: 10.1128/jvi.00674-26 (PMC13386967; doi:10.1128/jvi.00674-26)

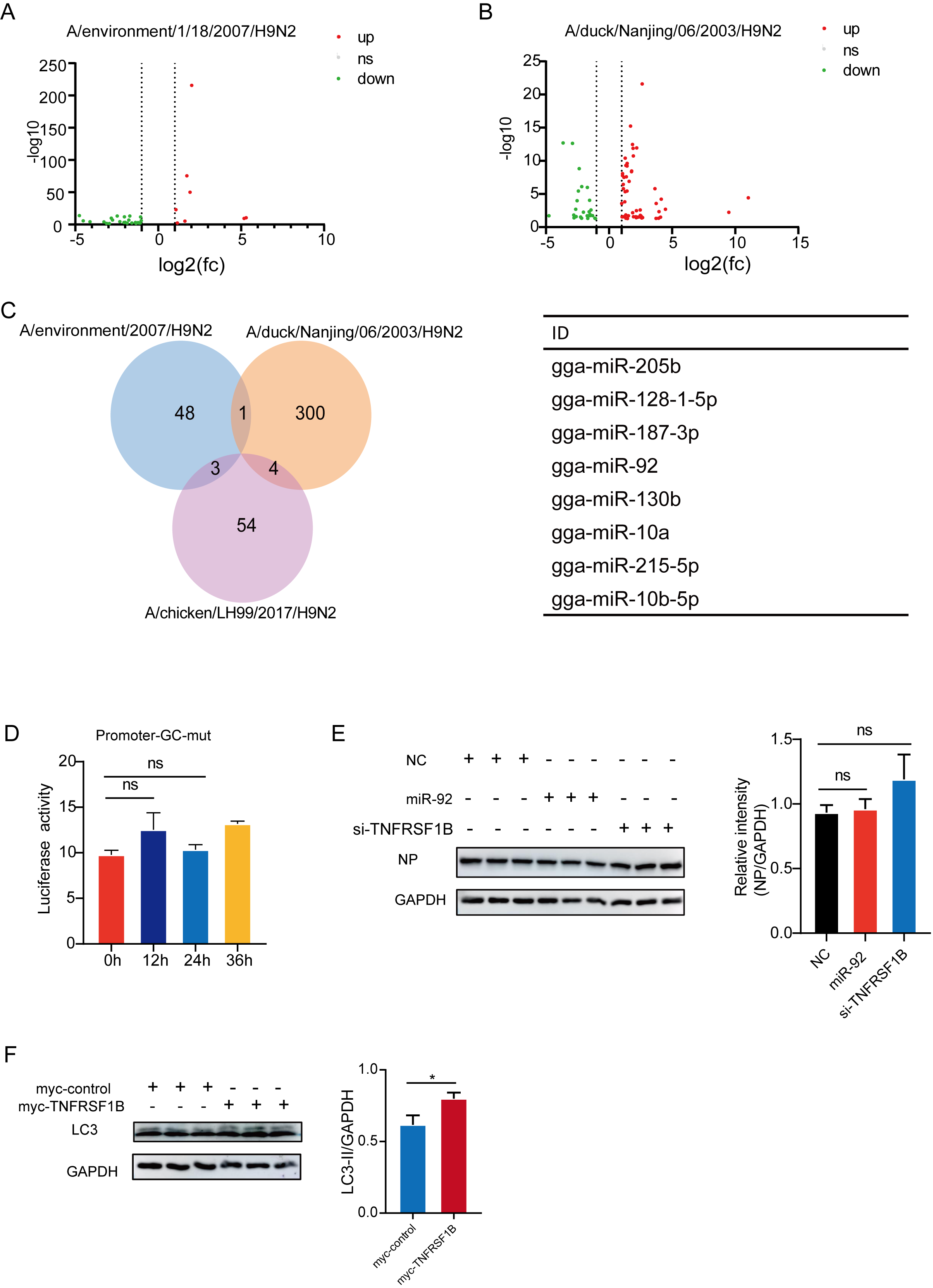

Supplement: Figure S1 — Analysis of miRNA profiles in avian cells infected with three H9N2 influenza A virus strains. [file jvi.00674-26-s0002.tif]

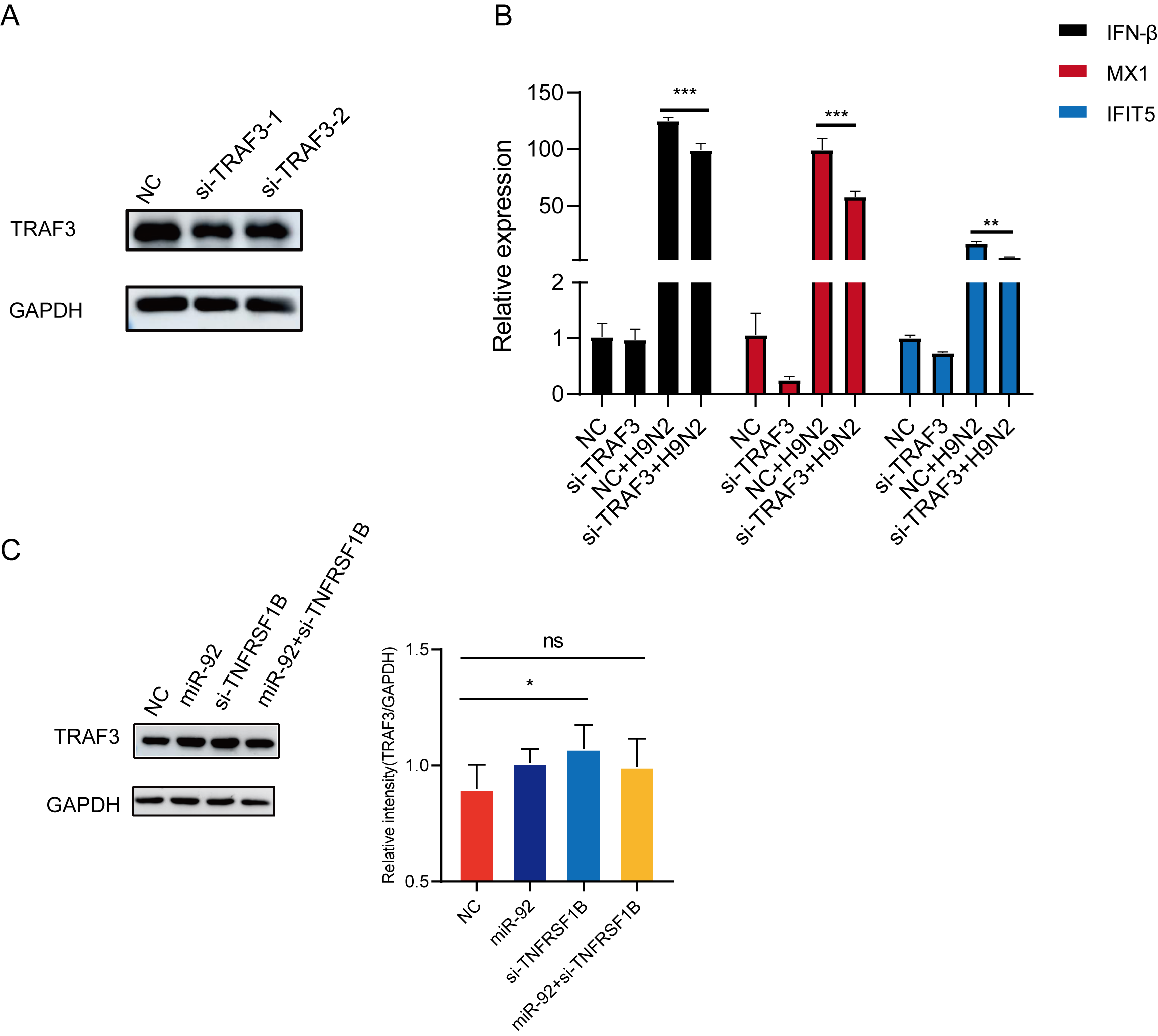

Supplement: Figure S2 — TRAF3 knockdown regulates interferon response upon H9N2 influenza virus infection. [file jvi.00674-26-s0003.tif]

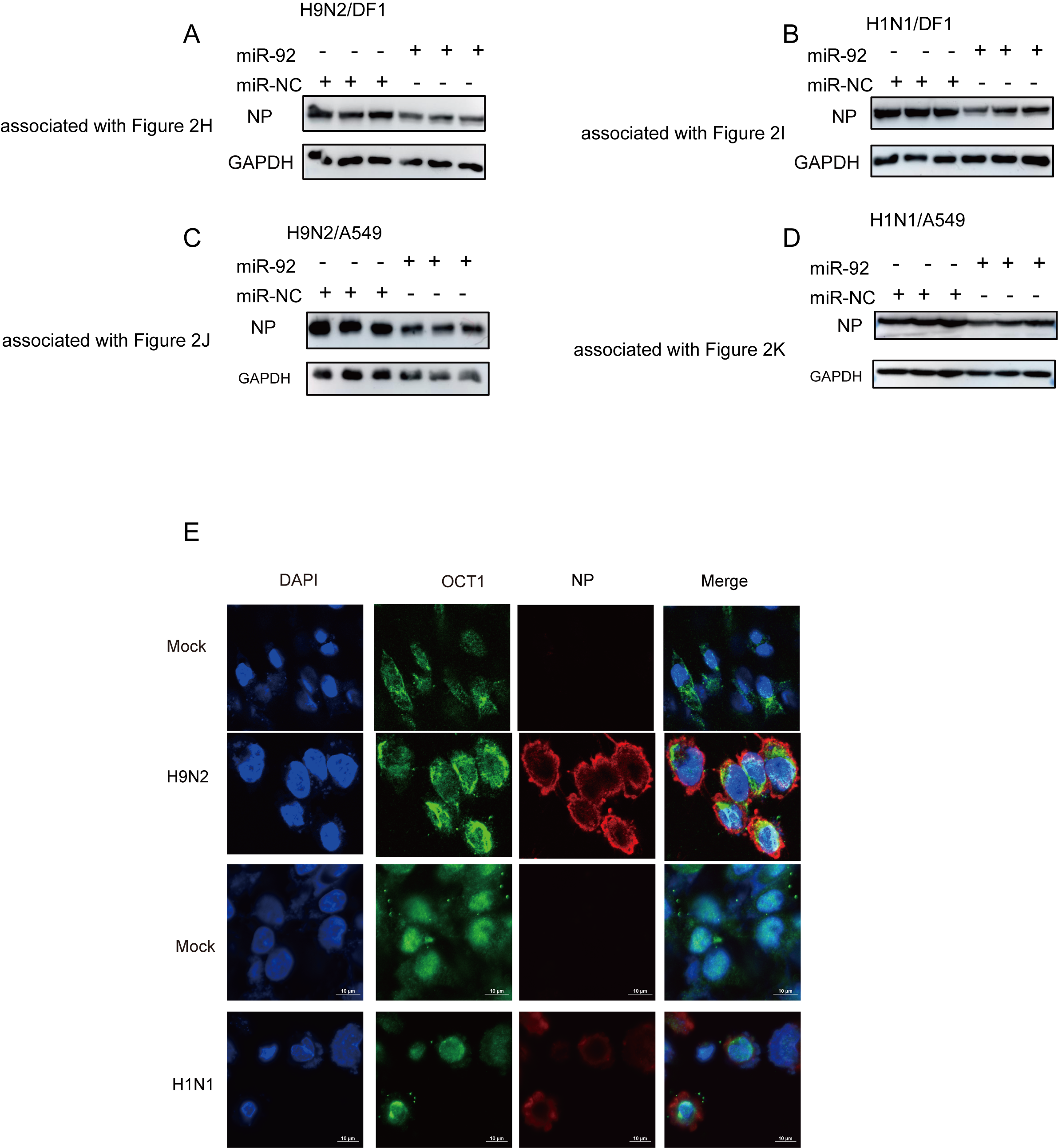

Supplement: Figure S3 — Quantitative analysis of Western blot results and subcellular localization of OCT1 upon influenza virus infection. [file jvi.00674-26-s0004.tif]

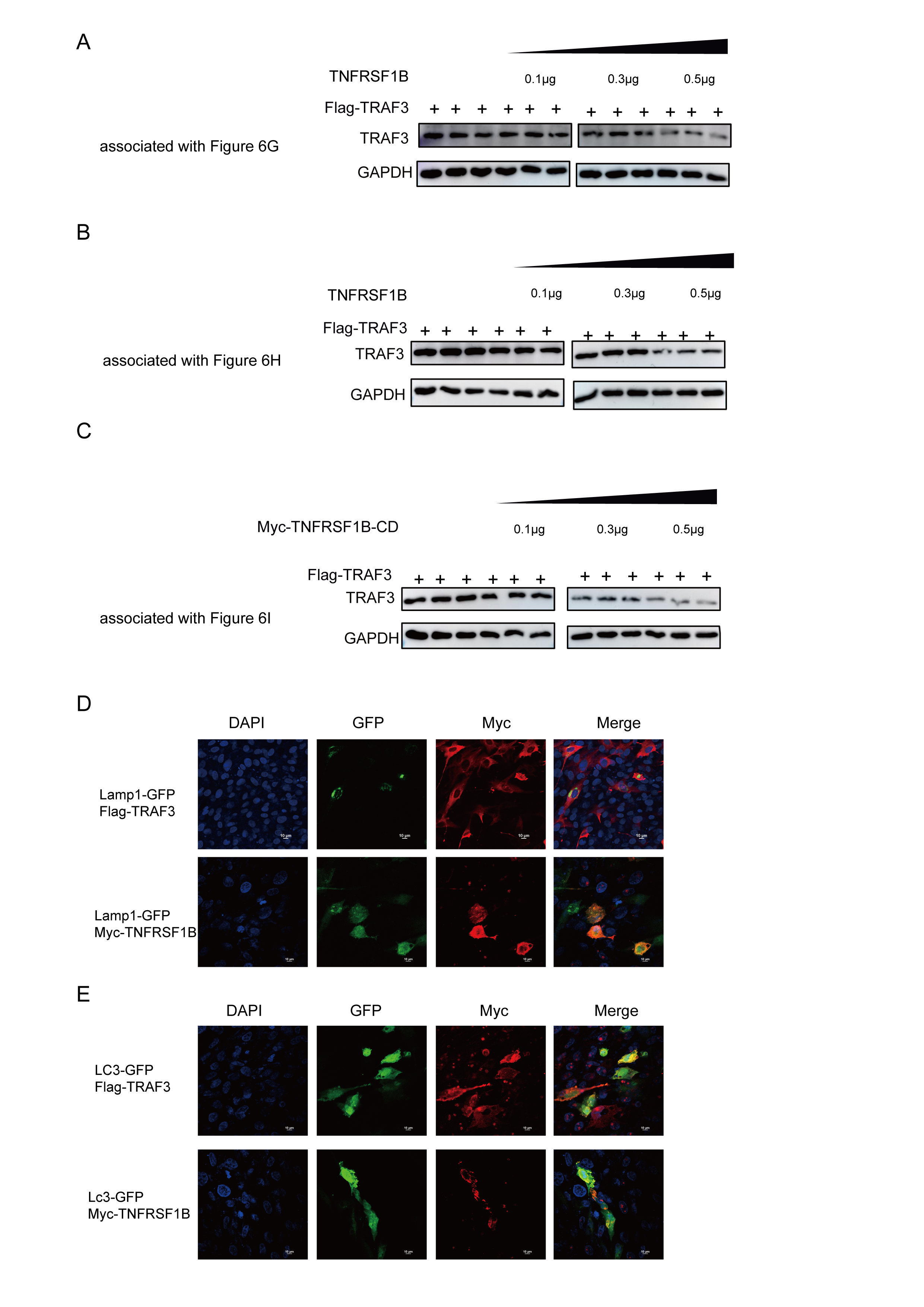

Supplement: Figure S4 — Quantification of Western blot results and co-localization analysis of TNFRSF1B and TRAF3 with lysosomal and autophagy markers. [file jvi.00674-26-s0005.tif]
