# Supplementary material for: Gga-miR-92-targeted TNFRSF1B inhibits the replication of influenza A virus by degrading TRAF3
Source: J Virol. 2026 Jun 12;100(7):e00674-26. doi: 10.1128/jvi.00674-26 (PMC13386967; doi:10.1128/jvi.00674-26)
Supplement: Supplemental legends — Legends for Fig. S1 to S4. [file jvi.00674-26-s0006.docx]

**Supplementary Figure 1**

(A) Differential miRNA profiling via high-throughput sequencing in avian cells infected with A/environment/1/18/2007/H9N2 virus. (B) Differential miRNA profiling via high-throughput sequencing in avian cells infected with A/duck/Nanjing/06/2003/H9N2 virus. (C) Venn intersection analysis of differentially expressed miRNAs across three H9N2 strains: A/environment/1/18/2007/H9N2, A/duck/Nanjing/06/2003/H9N2, and A/chicken/LH99/2017/H9N2. (D) The pGL3‑promoter‑GC‑mut reporter plasmid harboring a mutated GC promoter was constructed and transfected into DF1 cells. At 24 h post‑transfection, cells were infected with H9N2 avian influenza virus. Cells were harvested at various time points post‑infection to measure luciferase activity driven by the pGL3‑promoter‑GC‑mut construct. (E) DF1 cells were transfected with miR‑92 mimic or TNFRSF1B siRNA. At 24 h post‑transfection, cells were inoculated with H9N2 avian influenza virus at an MOI of 3. Virus binding was carried out on ice for 1 h, after which cells were washed three times with cold PBS (pH 7.4) to remove unbound virions. Viral growth medium was added, and cells were incubated at 37 °C for 1 h to allow viral internalization. Cells were then washed three times with cold PBS (pH 1.5) to remove non‑internalized virions, harvested in NP‑40 lysis buffer, and subjected to Western blotting for detection of viral nucleoprotein expression. (F) 293T cells were transfected with myc‑control or myc‑TNFRSF1B plasmid. At 24 h post‑transfection, cell lysates were analyzed by Western blotting to assess LC3‑II expression. Data shown are the mean ± SD from three independent experiments. Western blot results are representative of three independent experiments. All statistical analyses were performed using unpaired two‑tailed t-test . *, P < 0.05, **, P < 0.01, ***, P < 0.001.

**Supplementary Figure 2.**

(A) DF1 cells were transfected with TRAF3‑targeting siRNA, and cells were harvested 24 h post‑transfection for detection of TRAF3 protein expression by Western blotting. (B) DF1 cells were transfected with TRAF3‑targeting siRNA. At 24 h post‑transfection, cells were infected with H9N2 avian influenza virus at an MOI of 1. At 24 h post‑infection, cells were collected and the expression levels of IFN‑β and interferon‑stimulated genes (ISGs) were determined by quantitative real‑time PCR. (C) DF1 cells were transfected with miR‑92, TNFRSF1B siRNA, or co‑transfected with both miR‑92 and TNFRSF1B siRNA. At 24 h post‑transfection, cells were infected with H9N2 avian influenza virus, and TRAF3 protein expression was analyzed by Western blotting at 24 h post‑infection. Data shown are the mean ± SD from three independent experiments. Western blot results are representative of three independent experiments. All statistical analyses were performed using unpaired two‑tailed t-test or one-way ANOVA. *, P < 0.05, **, P < 0.01, ***, P < 0.001.

**Supplementary Figure 3.**

(A-D) Quantitative analysis of Western blot results from three independent biological replicates for Figure 2H–K. (E) DF1 cells were infected with H9N2 or H1N1 subtype influenza virus at an MOI of 1. At 24 hpi, OCT1 protein localization was detected by immunofluorescence assay.

**Supplementary Figure 4**

(A-C) Quantitative analysis of Western blot results from three independent biological replicates for Figure 6G–I. (D) DF‑1 cells were co‑transfected with MYC‑tagged TNFRSF1B‑ICD together with GFP‑tagged LAMP1, or FLAG‑tagged TRAF3 together with GFP‑tagged LAMP1. The co‑localization of the indicated proteins was examined by indirect immunofluorescence staining. (E) DF‑1 cells were co‑transfected with MYC‑tagged TNFRSF1B‑ICD and GFP‑tagged LC3, or FLAG‑tagged TRAF3 and GFP‑tagged LC3. The co‑localization of each protein pair was analyzed by immunofluorescence assay.
